# Soft tissue changes associated with Class III orthopaedic treatment in growing patients: a systematic review and meta-analysis

**DOI:** 10.1186/s40510-025-00558-2

**Published:** 2025-03-17

**Authors:** Ahmad Marwan Alhamwi, Ahmad Sharafeddin Burhan, Fehmieh Rafik Nawaya, Kinda Sultan

**Affiliations:** 1 Department of Orthodontics, University of Damascus Dental School, Damascus, Syrian Arab Republic; 2 Department of Pediatric Dentistry, Faculty of Dentistry, Syrian Private University., Damascus, Syrian Arab Republic

**Keywords:** Skeletal class III, Systematic review

## Abstract

**Introduction:**

Achieving a harmonious soft tissue profile and enhancing facial appearance are key goals of early treatment for skeletal class III malocclusion.

**Aim:**

To summarize the current evidence regarding the effects of Class III orthodontic treatment on facial soft tissues, and to compare various Class III orthodontic appliances.

**Methods:**

A comprehensive search was conducted up to July 2024, using seven databases, with no language restrictions. RCTs and controlled non-randomized studies were included in this systematic review. The GRADE framework was applied to evaluate the quality of evidence.

**Results:**

Thirty studies were included in this review, of which sixteen were appropriate for quantitative synthesis. The age range fell between 6.6 and 12.3 years. The FM/RME protocol resulted in a 1.58 mm increase in upper lip protrusion and a 4.73-degree decrease in the nasolabial angle compared to the control group. Chincup treatment led to a 2.13 mm increase in upper lip protrusion and a 2.63 mm decrease in lower lip protrusion compared to the control group. The pooled estimate demonstrated a significant increase of 1.82 mm in upper lip protrusion, a significant retrusion of 3.14 mm in the lower lip, and a backward movement of the chin by 4.8 mm in patients treated with miniplate-anchored orthopaedic facemask (FM/MP) compared to the untreated group. However, no significant difference was found between FM/RME and FM/MP, except for a noticeable decrease in the nasolabial angle in the FM/RME group. The analysis of FM/Alt-RAMEC versus FM/RME did not reveal any difference in soft tissue outcomes, except for the upper lip protrusion. The Alt-RAMEC group showed a more pronounced anterior movement of the upper lip by 0.67 mm compared to the RME group. The quality of evidence supporting these findings ranged from low to moderate.

**Conclusions:**

There is low to moderate evidence suggesting that early treatment positively influences the soft tissues in Class III patients. However, these conclusions are based on a two-dimensional analysis of cephalometric images, which may not provide complete or accurate information. Therefore, more RCTs using comprehensive 3D analysis are needed to confirm these results.

**Registration:**

PROSPERO (CRD42024517924).

**Supplementary Information:**

The online version contains supplementary material available at 10.1186/s40510-025-00558-2.

## Introduction

Dentofacial deformities can significantly impact an individual’s quality of life, self-esteem, social behaviour, and public perception [1]. This can result in them being seen as less attractive, less successful, and less socially acceptable [2]. The face is a critical component of the body that determines overall attractiveness and self-esteem [2, 3]. Therefore, more research has been devoted to analyzing the impact of orthodontic interventions on facial appearance [4, 5]. As part of contemporary orthodontic treatment planning, soft tissue assessment has emerged as a crucial aspect, with many orthodontists considering unfavourable soft tissue outcomes as a failure [4].

Skeletal class III malocclusion is one of the most challenging malocclusions for clinicians to diagnose and correct due to the intricate treatment process involved and the high rate of relapse caused by unpredictable growth patterns of patients affected by this type of skeletal discrepancy [6–9]. The pathogenesis of this malocclusion involves maxillary retrognathism, mandibular prognathism, or a combination of both [10]. About two-thirds of skeletal Class III malocclusion cases are associated with maxillary dysplasia [11]. Studies have suggested that early intervention is beneficial in developing Class III malocclusion cases. The main advantage of early Class III malocclusion treatment is to reduce the complexity of the second phase of treatment, it may also represent a valuable opportunity to avoid or minimize the likelihood of requiring orthognathic surgery in late adolescence [12, 13].

Over the years, various orthopaedic appliances and treatment techniques have emerged to address this type of malocclusion including, facemask, reverse headgear, chincup, Frankel III functional appliance, Reverse Twin-Block (RTB), orthodontic removable traction appliance (ORTA), and the bone-anchored maxillary protraction appliances [14]. Among these options, many clinicians prefer the facemask appliance to address a retrognathic maxilla. Conversely, the chincup is thought to redirect the growth of a prognathic mandible [14].

Skeletal class III patients with a concave facial profile, a retruded nasomaxillary area, and a protrusive lower face and lips are more concerned about their facial appearance than their occlusion [9, 15]. The main goal of class III malocclusion treatment should be to achieve a harmonious soft tissue profile and improve the overall facial appearance rather than focusing solely on occlusion like with other malocclusion types [16, 17]. However, many studies have reported that early orthopaedic treatment can result in favourable soft tissue changes for skeletal Class III malocclusion patients [6, 18, 19].

Numerous systematic reviews have been published to examine the dentoskeletal effects of this type of therapy in growing subjects with Class III disharmonies. Still, no systematic review has been published mainly aiming to critically and systematically appraise the available evidence regarding the facial soft tissue alterations associated with different Class III orthopaedic appliances. Therefore, the focused question of this review was ‘In growing skeletal Class III patients, does orthopaedic treatment enhance soft tissue outcomes?’

## Materials and methods

### Protocol and registration

This systematic review was conducted and reported in advance by strictly adhering to the Preferred Reporting Items for Systematic Reviews and Meta-Analyses (PRISMA) guidelines. During the first stages, the protocol of this review was written and registered in the PROSPERO Protocol Registry with the unique number CRD42024517924. More details of the protocol are available at https://www.crd.york.ac.uk/prospero/display_record.php?ID=CRD42024517924.

### Eligibility criteria

The eligibility criteria were defined according to the (PICOS) statement as follows:

(P) Participants: Pre-pubertal growing patients aged 7 to 12 years with a skeletal Class III malocclusion.

(I) Intervention: Orthodontic treatment with an orthopaedic appliance for early correction of skeletal Class III malocclusion.

(C) Comparison: Untreated control group, or intervention with the same appliance with different forces, mechanics, or appliances.

(O) Outcomes: Facial soft tissue changes detected via either 2D or 3D quantitative analysis of the measurements.

(S) Study design: Randomized controlled trials (RCTs) and non-randomized studies were chosen.

### Exclusion criteria

The following types of studies were excluded from the review: animal studies, in vitro studies, case reports or case series, finite element analysis studies, review articles, editorials, and non-clinical studies. Additionally, studies related to patients with cleft lip and palate or craniofacial syndromes, and studies with only one group, were excluded.

### Information sources and search

An extensive electronic search was accomplished independently from inception up to July 2024 in the following databases: the Cochrane Central Register of Controlled Trials (CENTRAL), PubMed, Scopus, Web of Science, Trip, EMBASE via OVID and ProQuest. No language or publication date limitations were applied. Bibliographies of the included articles and relevant systematic reviews were checked for further probable references. Ongoing or unpublished trials were also reviewed electronically through the World Health Organization’s International Clinical Trials Registry Platform (ICTRP) and ClinicalTrials.gov. Supplementary Table 1 illustrates more details of the electronic search strategy.

### Study selection and data extraction

Three reviewers were involved in the study selection process (A.M.A., A.S.B., and F.R.N.). The selection process was initiated by removing duplicate studies by two.

independent observers (A.M.A. and A.S.B.) then, studies were screened by titles and abstracts. Finally, the full text of all remaining papers was assessed using the predefined eligibility criteria. Studies that did not meet one or more inclusion criteria were excluded. The two reviewers’ level of agreement was analyzed using Cohen’s kappa statistics. Discrepancies were resolved through discussions with a third author (F.R.N.). Details regarding the author, year, sample size, participants, age, groups, outcome assessment, and results were extracted from the selected articles and organized into customized tables. Data extraction was separately achieved by two researchers (A.M.A. and A.S.B.), and any disagreements were resolved by discussion with the help of a third author (F.R.N.) to reach the final decision.

### Assessment of risk of bias in individual studies

The quality of the included articles was assessed by two independent reviewers (A.M.A. and A.S.B.) using The Revised Version of Cochrane’s Risk of Bias (ROB2) tool [20]. Any conflict was resolved by discussion with a third author. Risk of bias was evaluated (low, some concerns, and high) for the following domains: bias arising from the randomization process, bias due to deviations from intended interventions, bias due to missing outcome data, bias in the measurement of the outcomes, and bias in the selection of the reported results. The judgement of the overall risk of bias was established using the following criteria:

- Low, if the study was judged to be at low risk of bias for all domains;
- Some concerns, if at least one or more domains were evaluated as at some concerns of bias but no domain was judged to be at high risk of bias;
- High, if at least one domain was assessed as high risk of bias.

For non-randomized studies, the Risk of Bias in Non-Randomized Studies of Intervention (ROBINS-I) tool was applied [19]. This tool assesses various domains for potential low, moderate, serious, and critical risk or no information such as confounding bias, participant selection bias, intervention classification bias, deviations in intended interventions, missing data bias, outcome measurement bias, and reported result selection bias. The researchers employed the Risk of Bias Visualization tool (ROBVIS) to visualize the risk of bias assessment [21].

### Risk of bias assessment across studies

The strength of evidence for each outcome was estimated by the Grading of Recommendations Assessment, Development, and Evaluation (GRADE) assessment [22]. For each outcome studied, the GRADE approach assesses the number of studies included, risk of bias, inconsistency of results, indirectness of evidence, and imprecision of results. Two observers carried out this analysis independently. Disagreements were resolved via discussion with the third author.

### Summary measures, approach to synthesis and analysis

Review Manager software (RevMan) version 5.4 (The Nordic Cochrane Centre, The Cochrane Collaboration; Copenhagen, Denmark) was utilized to analyse the collected data. The included studies were evaluated for heterogeneity both clinically and statistically. Clinical heterogeneity was determined by comparing the treatment protocols used, including participant characteristics, eligibility criteria, interventions, and outcomes. Statistical heterogeneity was assessed using the chi-square test, where a *P*-value of less than 0.01 was considered significant. The I^2^ index was also employed to describe the degree of heterogeneity across the studies [23]. Standardized mean differences (SMDs) with their associated 95% CIs and the inverse variance method were chosen for the analysis method. The outcome measure was weighted (weighted mean difference, WMD) using calculations based on a random or fixed effects model. A qualitative synthesis was utilised for outcomes that were assessed by a single study or by different outcome measures among studies.

## Results

### Literature search flow

**Fig. 1:**
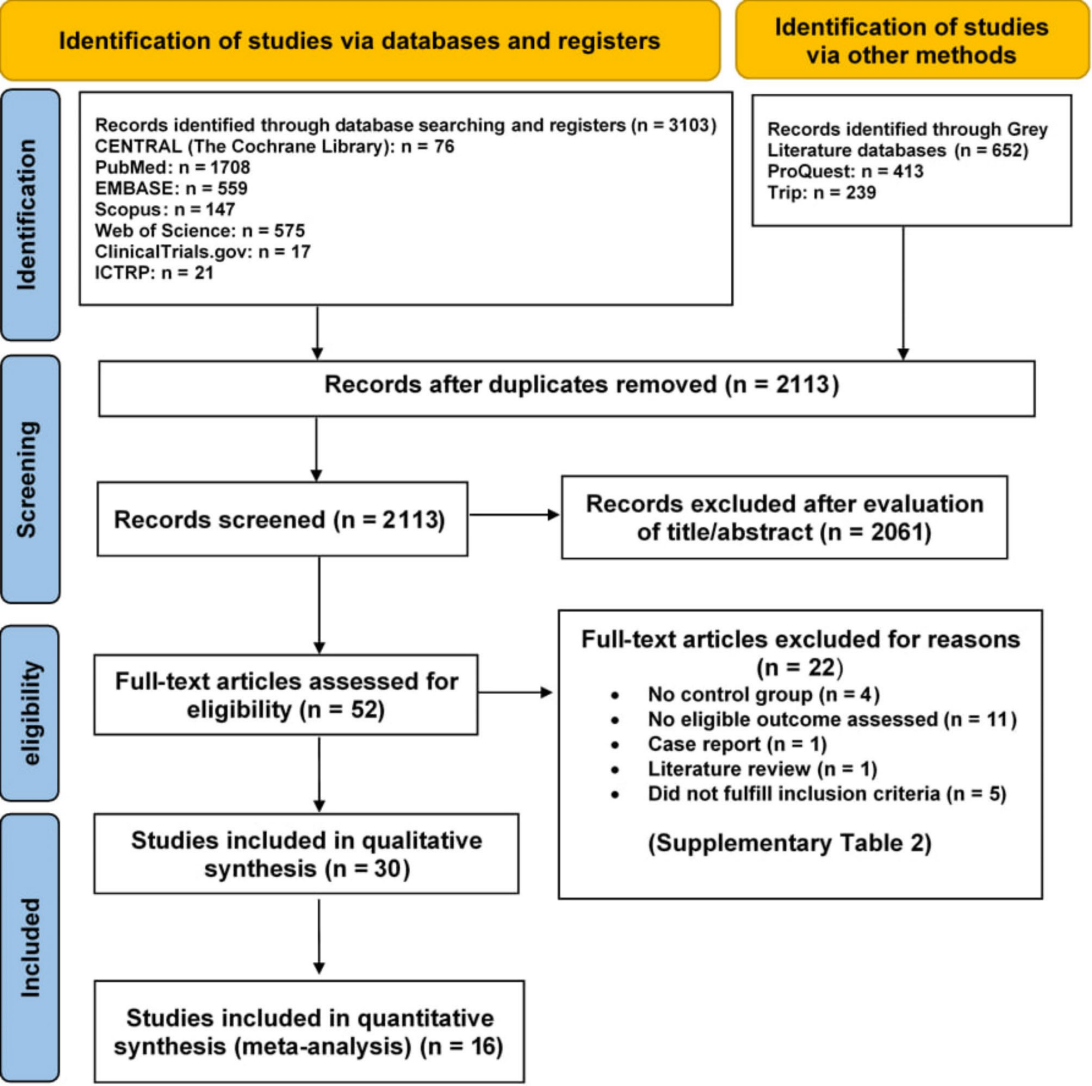
PRISMA flow diagram of the included studies

Upon conducting the initial search of the database, a total of 3755 articles were identified. After eliminating duplicates and screening the titles and abstracts of the identified citations, the inclusion and exclusion criteria were applied to the 53 full-text references. The agreement between the reviewers showed a high level of reliability, (K = 0.92). Excluded articles after full-text evaluation, and the reasons for exclusion, are presented in Supplementary Table 2. Ultimately, 30 studies met our criteria and were selected for this systematic review. Of these, 16 studies were incorporated into the quantitative synthesis (meta-analysis). The screening and selection methodology is visualized in Fig. 1, following the PRISMA flow chart.

### Characteristics of the included studies

Table 1 summarises the characteristics of the thirty studies included in this review. The studies were published in English between 1998 and 2023, eight of which were RCTs, while the other four were non-randomized studies. The studies were geographically diverse, with thirteen conducted in Turkey, four in South Korea, three in India, two in Syria, China and Egypt, one in the United States and one in Spain. A total of 1292 patients were included in the thirty studies. The age range fell between 6.6 and 12.3 years. The total sample size averaged 18-to-67 patients per study, all of them were growing patients. Class III malocclusion was defined by both skeletal and dental parameters. Various appliances were used in the studies mentioned, including chincup (CC), facemask (FM), or FM associated with rapid maxillary expansion (FM/RME), with alternate rapid maxillary expansion and constriction (Alt-RAMEC + FM), with bionator III (FM + Bio), with miniplates (FM/MP) or with splints (FM + splint) in at least one group. Additionally, orthodontic removable traction appliance (ORTA), removable mandibular retractor (RMR) appliance, novel magnetic orthopaedic appliance (MOA-III), intermaxillary Class III elastics (C3E) anchored by a hybrid hyrax (HH), reverse forsus, and miniscrew-anchored inverted forsus.

**Table 1:** Characteristics of the included studies

Study/setting	Study design	Number of participants (M/F)	Inclusion criteria	Treatment	Pre-treatment age (years)	Assessment method	Outcomes
Ağlarcı 2016 [42] Türkiye	NRS	Group 1: 25 (12/13) Group 2: 25 (12/13)	skeletal class III malocclusion characterized by maxillary deficiency and/or mandibular protrusion, ANB° < 0, Wits < 1, retrusive nasomaxillary complex, anterior crossbite, and concave profile; positive overbite.	Group 1: FM Group 2: Skeletal-anchorage therapy with mini-plates.	Group 1: 11.2 Group 2: 11.7	Ceph	- Upper lip position. - Lower lip position. - Nasolabial angle.
Akbulut 2023 [37] Türkiye	NRS	Group 1: 15 (10/5) Group 2: 15 (10/5)	skeletal Class III malocclusion with negative Wits values, and 0 mm or less over-jet.	Group 1: FM + RME Group 2: FM + Alt-RAMEC	Group 1: 11.6 Group 2: 10.9	Ceph	- Upper lip position. - Lower lip position. - Nasolabial angle.
Akin 2015 [24] Türkiye	NRS	Group 1: 25 (10/15) Group 2: 25 (9/16) Group 3: 17 (8/9)	Class III molar relationship, Edge-to-edge or anterior crossbite relationship, ANB angle of 0° or less.	Group 1: FM/RPE Group 2: CC Group 3: untreated	Group 1: 10.3 Group 2: 9.8 Group 3: 10.1	Ceph	- Upper lip position. - Lower lip position.
Alarcón 2015 [25] Spain	NRS	Group 1: 42 (21/21) Group 2: 25 (12/13)	skeletal Class III malocclusion (ANB angle < 0° and Wits appraisal ≤ 2 mm) due to mandibular prognathism (SNB angle > 82°) with normal maxilla (SNA angle = 82°±2°), molar Class III of at least half a cusp, anterior cross-bite or edge-to-edge incisal relationship.	Group 1: CC Group 2: untreated	Group 1: 8.5 Group 2: 8.5	Ceph	- Upper lip position. - Lower lip position. - Nasolabial angle. - Soft tissue pogonion (Pg’). - Profile facial angle. - Upper lip thickness. - Lower lip thickness.
Alzabibi 2021 [6] Syria	RCT	Group 1: 21 (12/9) Group 2: 19 (8/11)	skeletal Class III malocclusion, -3° ≤ ANB ≤ 1.5°, -3 mm ≥ Wits appraisal ≥ -9 mm, patients with mixed dentition.	Group 1: ORTA appliance Group 2: untreated	Group 1: 8.95 Group 2: 9.14	Ceph	- Upper lip position. - Lower lip position. - Nasolabial angle. - Mentolabial angle.
Buyukcavus 2020 [38] Türkiye	NRS	Group 1: 18 (8/10) Group 2: 19 (9/10) Group 3: 18 (9/9)	Negative overjet, maxillary deficiency, concave profile, decreased SNA angle, and negative ANB angle.	Group 1: FM/RME Group 2: FM/ Alt-RAMEC Group 3: FM/MP	Group 1: 10.5 Group 2: 11.6 Group 3: 11.9	Ceph	- Upper lip position. - Lower lip position. - Nasolabial angle. - Soft tissue pogonion (Pg’).
Canturk and Celikoglu 2015 [41] Türkiye	NRS	Group 1: 15 (8/7) Group 2: 15 (6/9)	skeletal Class III malocclusion (ANB angle < 0°) and negative overjet; vertically normal growth pattern, no signs of functional Class III malocclusion.	Group 1: FM started after the completion of the Alt-RAMEC Group 2: FM started simultaneously with the Alt-RAMEC	Group 1: 11.2 Group 2: 10.5	Ceph	- Upper lip position. - Lower lip position. - Soft tissue pogonion (Pg’). - Profile facial angle.
Celikoglu 2015 [43] Türkiye	RCT	Group 1: 16 (6/10) Group 2: 16 (7/9)	skeletal class III malocclusion (ANB angle < 0°) and negative overjet, vertically normal growth pattern, no signs of functional class III malocclusion.	Group 1: FM + RME Group 2: MMP	Group 1: 12 Group 2: 11.5	Ceph	- Upper lip position. - Lower lip position. - Nasolabial angle. - Profile facial angle.
Cozza 2004 [5] Italy	NRS	Group 1: 30 (17/13) Group 2: 24 (14/10)	skeletal Class III relationship caused by maxillary retrognathism without other craniofacial anomalies.	Group 1: FM + BIO Group 2: untreated.	Group 1: 6.8 Group 2: 6.9	Ceph	- Upper lip position. - Lower lip position. - Nasolabial angle. - Profile facial angle. - Upper lip thickness. - Upper lip strain.
Eissa 2018 [26] Egypt	NRS	Group 1: 16 (7/9) Group 2: 16 (8/8)	skeletal Class III malocclusion, -4 < ANB < 0, maxillary deficiency (SNA < 78) with or without mandibular prognathism, Angle Class III molar relationship with or without anterior crossbite.	Group 1: miniscrew-anchored inverted FRD Group 2: untreated	Group 1: 12.3 Group 2: 11.9	Ceph	- Upper lip position. - Lower lip position. - Nasolabial angle.
James 2020 [44] India	RCT	Group 1: 26 (10/16) Group 2: 26 (14/12)	skeletal Class III with maxillary retrusion, ANB < 0, negative or zero overjet, and Class III molar relationship with no signs of functional Class III malocclusion	Group 1: FM + Alt-RAMEC Group 2: FM + Alt-RAMEC + Class III Elastic	Group 1: 10 Group 2: 10.2	Ceph	- Upper lip position. - Lower lip position. - Nasolabial angle. - Soft tissue pogonion (Pg’).
Jang 2021 [48] South Korea	NRS	Group 1: 31 (14/17) Group 2: 36 (22/14)	Negative overjet or edge-to-edge bite; ANB < 0˚, Wits appraisal< -4 mm.	Group 1: FM/MP Group 2: FM/RME	Group 1: 11.1 Group 2: 11	Ceph	- Lower lip position. - Nasolabial angle.
Kamel 2023 [27] Egypt	RCT	Group 1: 17 (9/8) Group 2: 13 (8/5)	skeletal Class III with maxillary retrusion, ANB < 0, Wits < − 2.	Group 1: intermaxillary Class III elastics (C3E) anchored by a hybrid hyrax (HH) in the maxilla and a bone-supported bar in the mandible Group 2: untreated	Group 1: 11.3 Group 2: 11.5	Ceph	- Upper lip position. - Lower lip position. - Soft tissue pogonion (Pg’).
Kiliçoğlu and Kirliç 1998 [28] Türkiye	RCT	Group 1: 16 (0/16) Group 2: 10 (0/10)	Skeletal Class III with maxillary retrognathism, ANB angle less than − 1, Angle Class III with anterior crossbite.	Group 1: FM Group 2: untreated	Group 1: 8.6 Group 2: 9.2	Ceph	- Upper lip position. - Lower lip position. - Nasolabial angle. - Mentolabial angle. - Profile facial angle. - Soft tissue pogonion (Pg’). - Upper lip thickness. - Upper lip strain.
Lee 2022 [46] South Korea	NRS	Group 1: 20 (7/13) Group 2: 20 (9/11)	skeletal and dental Class III malocclusion with retrognathic maxilla, ANB < 1, anterior crossbite to edge-to-edge bite.	Group 1: FM + MP Group 2: FM + RME	Group 1: 10.5 Group 2: 10	Ceph	- Upper lip position. - Lower lip position. - Nasolabial angle.
Lee 2012 [45] South Korea	NRS	Group 1: 10 (5/5) Group 2: 10 (4/6)	skeletal and dental Class III malocclusion with maxillary hypoplasia, SNA < 80; ANB < -1, anterior crossbite and positive overbite.	Group 1: FM/MP Group 2: FM/RME	Group 1: 11.2 Group 2: 10.7	Ceph	- Upper lip position. - Lower lip position.
Lim 2021 [47] South Korea	NRS	Group 1: 25 (15/10) Group 2: 25 (13/12)	skeletal Class III patients with anterior cross-bite and Class III molar relationship.	Group 1: creative horseshoe appliance (CHS) with two Class III elastics Group 2: Petit-type facemask	Group 1: 8.6 Group 2: 8.9	Ceph	- Lower lip position. - Nasolabial angle.
Liu 2020 [40] China	NRS	Group 1: 20 (14/6) Group 2: 20 (9/11)	Class III molar relationship, anterior crossbite, maxillary retrognathism, ANB < 0	Group 1: FM/ Banded appliance Group 2: FM/ Modified appliance	Group 1: 8.35 Group 2: 8.65	Ceph	- Upper lip position. - Lower lip position. - Soft tissue pogonion (Pg’).
Ozbilen 2023 [49]	NRS	Group 1: 16 (NR) Group 2: 16 (NR) Group 3: 16 (NR)	anterior crossbite, skeletal class III malocclusion with maxillary deficiency (SNA < 80 °, ANB < 2).	Group 1: FM + RME Group 2: FM + Alt-RAMEC Group 3: untreated	Group 1: 9.94 Group 2: 9.74 Group 3: 9.46	3D stereophotogrammetry	- Upper lip - Lower lip and chin
Parayaruthottam 2018 [39] India	NRS	Group 1: 9 (NR) Group 2: 9 (NR)	skeletal Class III malocclusion (ANB < 0°) due to a retrognathic maxilla with or without associated mandibular prognathism	Group 1: FM + RME Group 2: FM + Alt-RAMEC	Group 1: 10.1 Group 2: 10.3	Ceph	- Upper lip position. - Lower lip position. - Nasolabial angle. - Soft tissue pogonion (Pg’).
Pavoni 2019 [29] Italy	NRS	Group 1: 32 (17/15) Group 2: 20 (10/10)	Class III malocclusion with a Wits appraisal of − 2 mm or less, anterior crossbite or incisor end-to-end relationship.	Group 1: FM/RPE Group 2: untreated	Group 1: 8.4 Group 2: 8.7	Ceph	- Upper lip position. - Lower lip position. - Nasolabial angle. - Profile facial angle.
Saleh 2013 [30] Syria	RCT	Group 1: 33 (17/16) Group 2: 34 (15/19)	skeletal Class III malocclusion, Class III molar relationship with anterior crossbite.	Group 1: RMR appliance Group 2: untreated	Group 1: 7.5 Group 2: 7.3	Ceph	- Upper lip position. - Lower lip position. - Nasolabial angle. - Profile facial angle. - Soft tissue pogonion (Pg’). - Mentolabial angle.
Şar 2014 [32] Türkiye	NRS	Group 1: 17 (NR) Group 2: 17 (NR) Group 3: 17 (NR)	Skeletal and dental Class III malocclusion with maxillary deficiency, ANB < 0, and retrusive nasomaxillary complex with or without mandibular prognathia, anterior crossbite and Angle Class III molar relationship.	Group 1: FM + MP Group 2: EL + MP Group 3: untreated	Group 1: 11.2 Group 2: 11.2 Group 3: 9.9	Ceph	- Upper lip position. - Lower lip position. - Soft tissue pogonion (Pg’).
Şar 2011 [31] Türkiye	NRS	Group 1: 15 (10/5) Group 2: 15 (8/7) Group 3: 15 (7/8)	Skeletal and dental Class III malocclusion with maxillary deficiency, ANB < 0, Wits appraisal < − 2 mm, anterior crossbite.	Group 1: FM/MP Group 2: FM Group 3: untreated.	Group 1: 10.9 Group 2: 10.3 Group 3: 10	Ceph	- Upper lip position. - Lower lip position. - Soft tissue pogonion (Pg’).
Sitaropoulou 2020 [50] Türkiye	NRS	Group 1: 20 (10/10) Group 2: 16 (10/6)	Class III skeletal relationship due to maxillary retrognathism; normal to low angle vertical growth pattern (SN-GoMe ≤ 32° ± 6); Wits appraisal less than − 1 mm	Group 1: FM/Alt-RAMEC Group 2: untreated	Group 1: 9.74 Group 2: 9.44	3D) photographs	- Labiale superior - Alar curvature - Subalare
Tripathi 2016 [8] India	NRS	Group 1: 10 (NR) Group 2: 10 (NR)	skeletal class III malocclusion with maxillary deficiency, ANB < 0, edge-to-edge bite, and normal or increased overbite.	Group 1: FM/MP Group 2: FM/RPE	Group 1: 10.1 Group 2: 9.9	Ceph	- Upper lip position. - Lower lip position. - Soft tissue pogonion (Pg’).
Üçem 2004 [35] Türkiye	NRS	Group 1: 14 (7/7) Group 2: 14 (7/7) Group 3: 14 (8/6)	Skeletal Class III malocclusions due to maxillary retrusion or a combination of maxillary retrusion and mandibular protrusion.	Group 1: DPA with 2 Class III elastics Group 2: FM Group 3: untreated	Group 1: 10.3 Group 2: 10.5 Group 3: 9.8	Ceph	- Lower lip position. - Nasolabial angle.
Vaughn 2005 [36] USA	RCT	Group 1: 15 (7/8) Group 2: 14 (7/7) Group 3: 17 (10/7)	Negative overjet, Class III molar relationship, ANB angle of 0° or less, Wits of 3 mm or more, and nasion perpendicular to A-point of 2 mm or less	Group 1: FM/RME Group 2: FM Group 3: untreated.	Group 1: 7.4 Group 2: 8.1 Group 3: 6.6	Ceph	- Nasolabial angle.
Yavan 2023 [33] Türkiye	RCT	Group 1: 15 (7/8) Group 2: 15 (9/6) Group 3: 15 (8/7)	Mild class III malocclusions caused by maxillary deficiency, ANB < 0°, Angle class III molar relation.	Group 1: FM/RPE Group 2: Reverse Forsus appliance Group 3: untreated	Group 1: 10.5 Group 2: 10.4 Group 3: 10.6	Ceph	- Upper lip position. - Lower lip position.
Zhao 2015 [34] China	NRS	Group 1: 36 (14/22) Group 2: 20 (9/11)	0°> ANB > -3°, Wits < 0 mm, Angle`s class III molar relationship with anterior cross-bite	Group 1: MOA-III Group 2: untreated	Group 1: 9.5 Group 2: 9.2	Ceph	- Upper lip position. - Lower lip position. - Profile facial angle.

Abbreviations: Alt-RAMEC: alternate rapid maxillary expansion and constriction, Ceph: cephalometric images, DPA: double-plate appliance, F: female, FM: facemask, M: male, FM/MP: miniplate-anchored facemask, EL: elastics, RMR: removable mandibular retractor, MOA-III: magnetic orthopaedic appliance, MMP: mini maxillary protractor, NR: not reported, NRS: non-randomized study, FM+BIO: Facemask + Bionator III, ORTA: orthodontic removable traction appliance, RCT: randomized clinical trial, RME: rapid maxillary expansion

### Risk of bias within studies

**Fig. 2:**
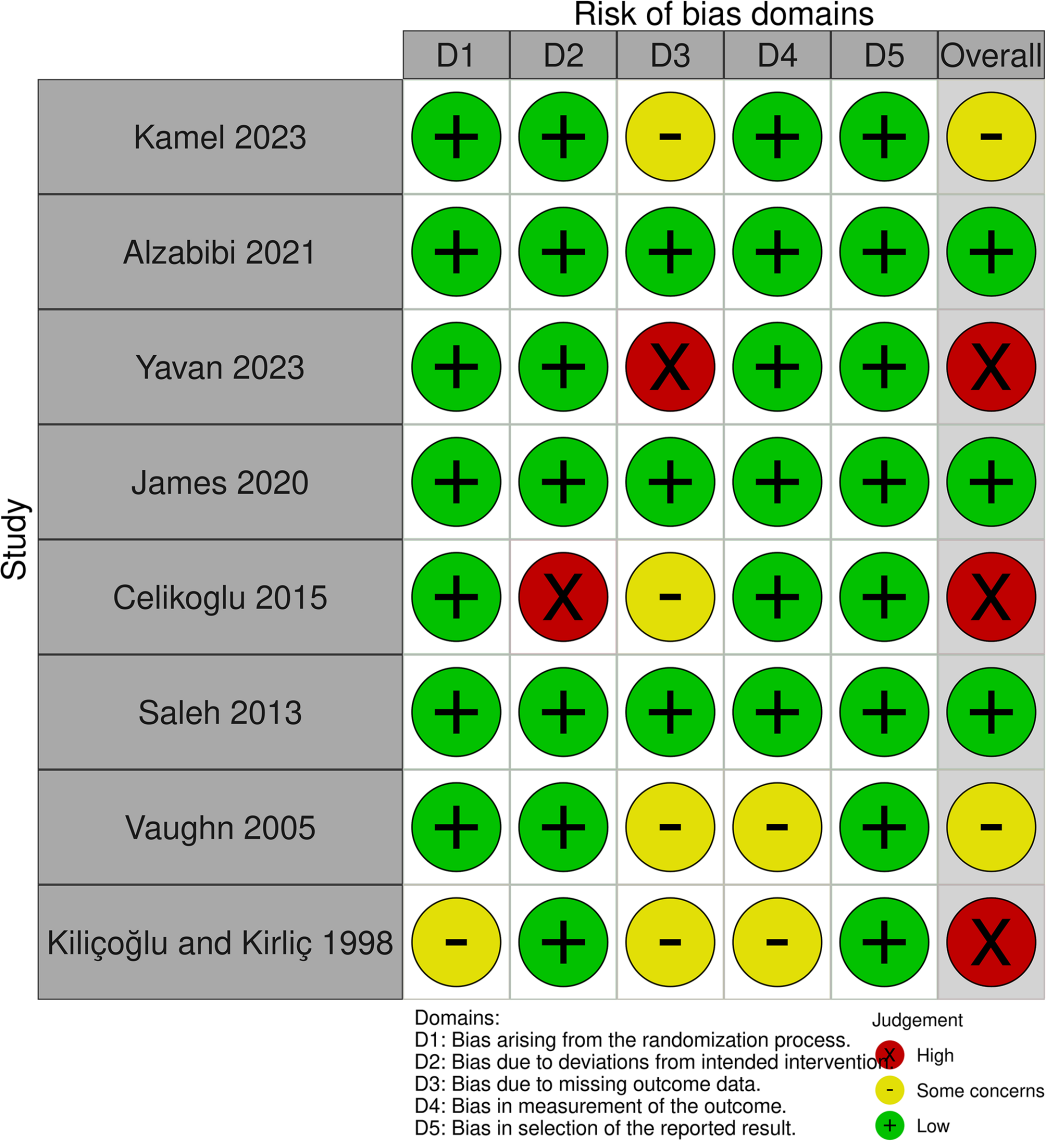
Summary of the risk of bias of randomized studies using ROB2 tool

**Fig. 3:**
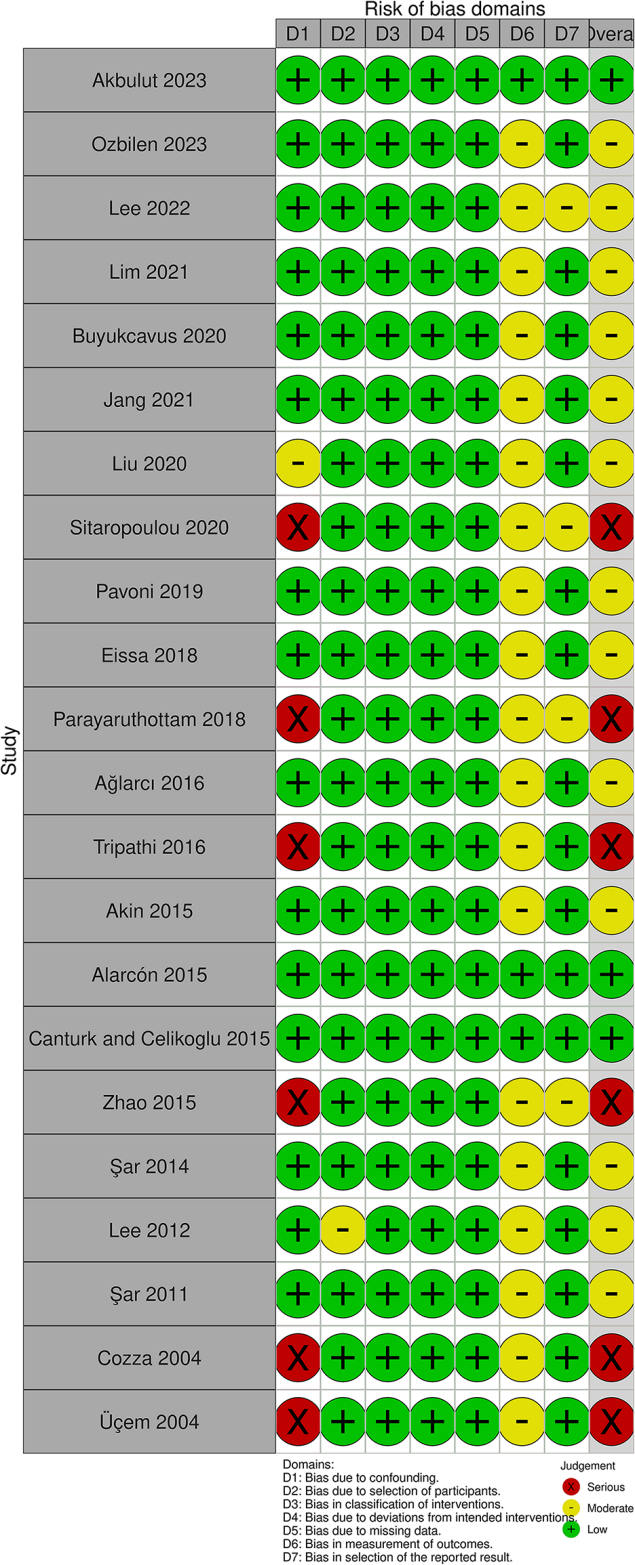
Summary of the risk of bias of non-randomized studies using ROBINS-I tool

A summary of the overall risk of bias assessment applied to the included studies is provided in Figs. 2 and 3. Three RCTs were judged to be at “high risk of bias”. Two RCTs were classified as having some concern of bias, whereas three RCTs were at low risk of bias. Using the ROBINS-I tool, 6 studies showed a serious risk of bias, 3 displayed a low risk of bias, and 13 articles were at moderate risk of bias. More details of the risk of bias assessment are shown in Supplementary Tables 3 and Supplementary Table 4.

### Effects of intervention

Each outcome measure’s mean and standard deviations were extracted from the included studies and organized in Supplementary Table 5. Based on the available studies, two distinct themes of comparisons could be made:

- Appliance vs. untreated control.
- Appliance 1 vs. Appliance 2.

### First: appliance vs. untreated control

Seventeen studies (6 RCT,11 CCT) examined the effects of orthopaedic appliances compared to an untreated control group. The outcomes mainly assessed were changes in upper lip protrusion, lower lip protrusion, nasolabial angle, mentolabial angle, and facial convexity. For the meta-analysis, data regarding these outcomes were only considered when at least two studies with the same orthopaedic appliance employed the same outcome measure.

#### Upper lip protrusion

**Fig. 4:**
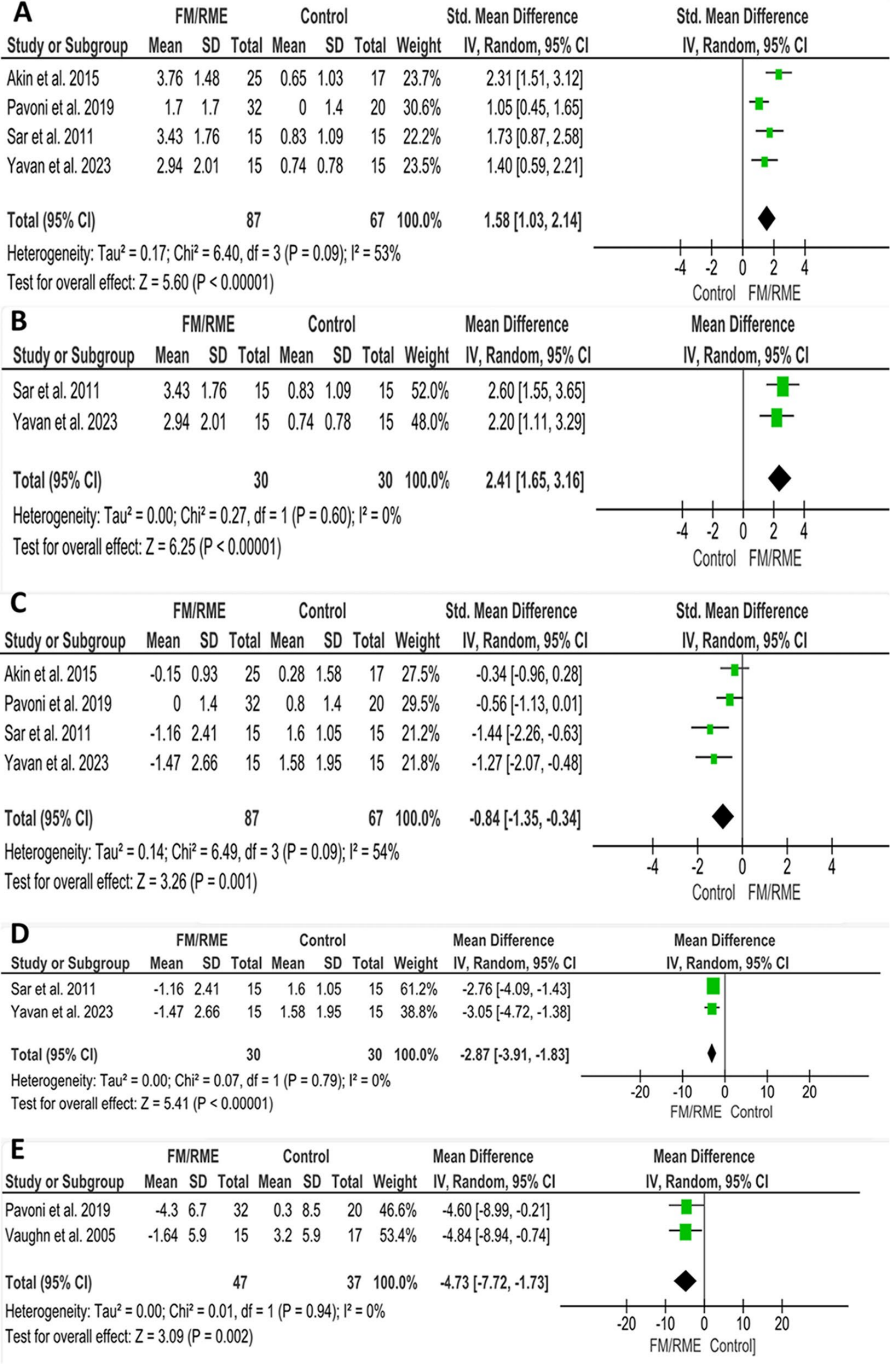
Forest plots of soft tissue changes associated with the FM/RME protocol compared to the control group: (**A**) Upper lip protrusion [SMD], (**B**) Upper lip protrusion [MD for studies assessed VR line], (**C**) Lower lip protrusion [SMD], (**D**) Lower lip protrusion [MD for studies assessed VR line], (**E**) Nasolabial angle

Eleven studies assessed this outcome [5, 6, 24–34]. Four studies [24, 29, 31, 33], comprising 154 patients (87 FM/RME, 67 untreated) looked at comparisons between facemasks associated with rapid maxillary expansion (FM/RME) and untreated control groups. Two studies [31, 33] used the vertical reference line (VRL) as a baseline, one study [29] measured the perpendicular distance between the upper lip’s anterior point and the subnasale-pogonion line, while another study [24] utilized the E line. Consequently, the standardized mean difference (SMD) was employed to combine these results effectively. The pooled estimate showed a statistically significant increase of 1.58 mm upper lip protrusion in patients treated with FM/RME compared to the control group. (SMD = 1.85; 95% CI (1.03, 2.14); *P* < 0.00001; Fig. 4A). The heterogeneity was moderate (Chi^2^ = 0.46; *P* = 0.09; I^2^ = 53%). According to GRADE, the overall strength of the evidence supporting this outcome was moderate Table 2. However, when combining the studies that used the vertical reference line (VRL) as a baseline [31, 33], the pooled estimate showed a statistically significant increase of 2.41 mm upper lip protrusion in patients treated with FM/RME compared to the control group. (MD = 2.41; 95% CI (1.65, 3.16); *P* < 0.00001; Fig. 4B).

**Table 2:** Summary of the findings according to the GRADE guidelines for the FM/RME vs. no treatment comparison

Certainty assessment						№ of patients	Effect on FM/RME group	Certainty	Comment
№ of studies	Risk of bias	Inconsistency	Indirectness	Imprecision	Other considerations		Absolute (95% CI)		
1- Upper lip protrusion (assessed with mm)									
4	serious	not serious	not serious	not serious	none	154	1.58 [1.03, 2.14]	⨁⨁⨁◯ Moderate^a^	Significant increase of 1.58 mm upper lip protrusion in patients treated with FM/RME compared to the control group (*P* < 0.00001)
2- Lower lip protrusion (assessed with mm)									
4	serious	not serious	not serious	not serious	none	154	-0.84 (-1.35;0.34)	⨁⨁⨁◯ Moderate^a^	Significant decrease of 0.84 mm lower lip protrusion in patients treated with FM/RME (*p* = 0.001)
3- Nasolabial angle (assessed with degrees)									
2	serious	not serious	not serious	not serious	none	84	-4.73 (-7.72, -1.73)	⨁⨁⨁◯ Moderate^a^	Significant decrease of 4.73 degrees in patients treated with FM/RME compared to the control group (*p* = 0.002)

a. Downgrade one level for risk of bias

Additionally, Kiliçoğlu and Kirliç [28] reported that patients treated with the FM appliance without expansion had a 2.6 mm increase in upper lip protrusion compared to an untreated control group.

**Fig. 5:**
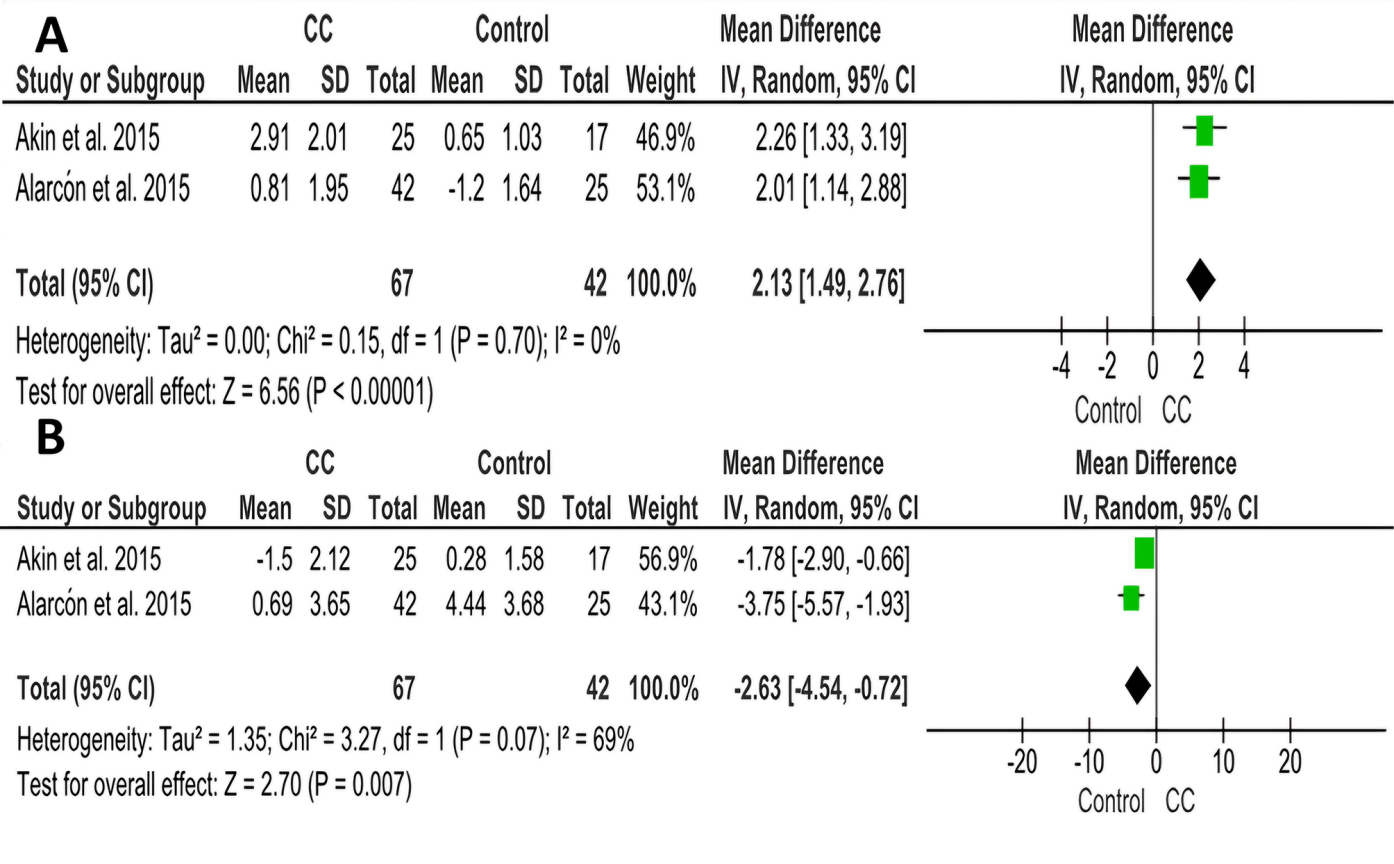
Forest plots of soft tissue changes associated with the chincup compared to the control group: (**A**) Upper lip protrusion, (**B**) Lower lip protrusion

Two of the scrutinised studies [24, 25] comprising 109 patients utilized the chincup compared to untreated patients with early skeletal Class III malocclusion treatment (67 chincups, 42 untreated). The pooled estimate showed a statistically significant increase of 2.13 mm upper lip protrusion in patients treated with CC compared to the control group. (MD = 2.13; 95% CI (1.49, 2.76); *P* < 0.00001; Fig. 5A). Heterogeneity was low (Chi^2^ = 0.15; *P* = 0.70; I^2^ = 0%). According to GRADE, the overall strength of the evidence supporting this outcome was moderate Table 3.

**Table 3:** Summary of the findings according to the GRADE guidelines for the CC vs. no treatment comparison

Certainty assessment						№ of patients	Effect on CA group	Certainty	Comment
№ of studies	Risk of bias	Inconsistency	Indirectness	Imprecision	Other considerations		Absolute (95% CI)		
1- Upper lip protrusion (assessed with mm)									
2	serious	not serious	not serious	not serious	none	109	2.13 [1.49, 2.76]	⨁⨁⨁◯ Moderate^a^	significant increase of 2.13 mm upper lip protrusion in patients treated with CC compared to the control group (*P* < 0.00001)
2- Lower lip protrusion (assessed with mm)									
2	serious	serious	not serious	not serious	none	109	-2.63 [-4.54, -0.72]	⨁⨁◯◯ Low^b^	significant decrease of 2.63 mm lower lip protrusion in patients treated with CC compared to the control group (*P* = 0.007)

a. Downgrade one level for risk of bias

b. Downgrade one level for risk of bias, and one level due to inconsistency

**Fig. 6:**
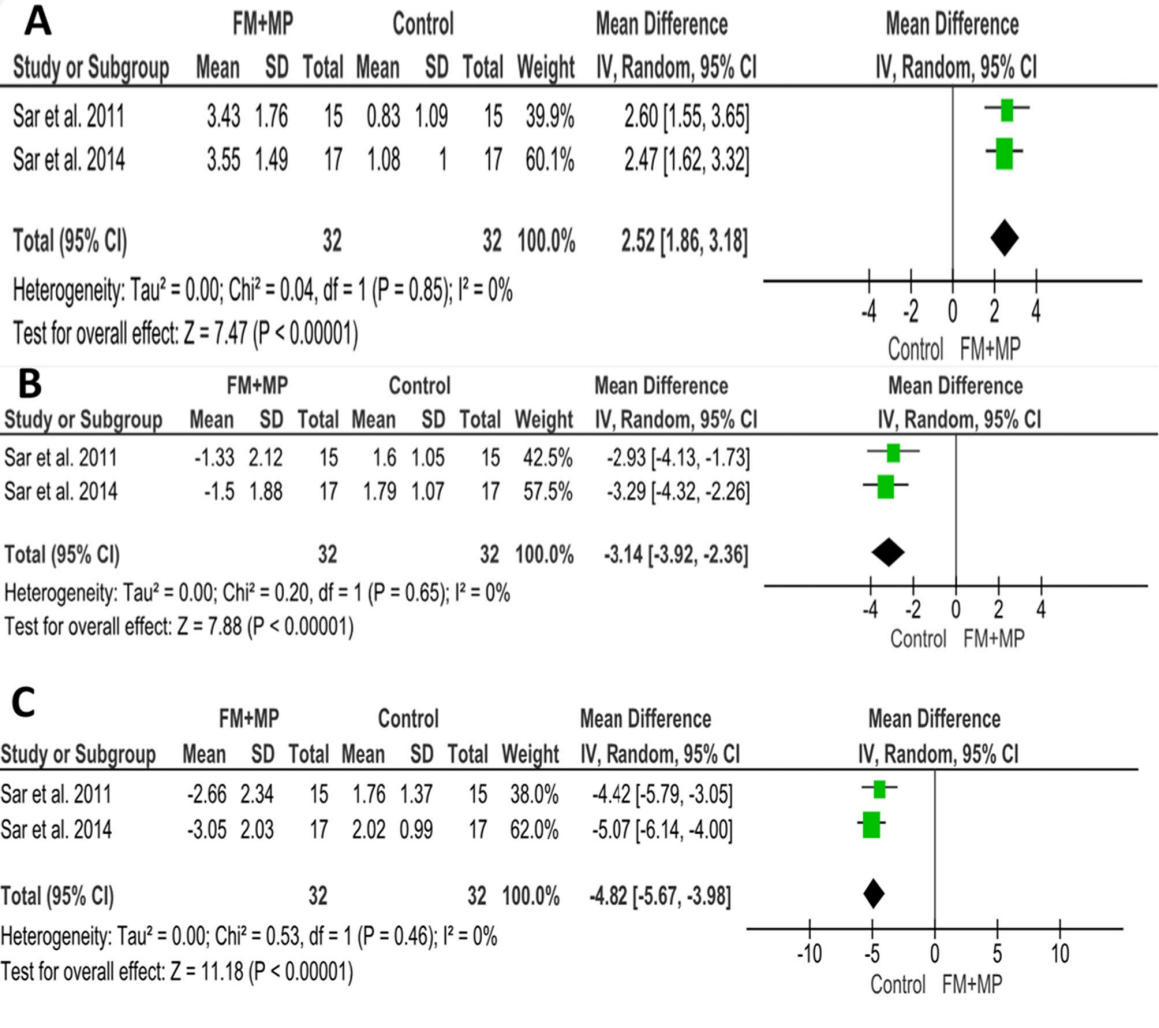
Forest plots of soft tissue changes associated with FM/MP compared to the control group: **A**) Upper lip protrusion, **B**) Lower lip protrusion, **C**) Soft tissue pogonion (Pg’)

Significant protrusion of upper lip was resulted with mean of 2.52 mm in patients treated with FM/MP compared to the control group. (MD = 2.52; 95% CI (1.86, 3.18); *P* < 0.00001; Fig. 6A). Heterogeneity was low (Chi^2^ = 0.04; *P* = 0.85; I^2^ = 0%). According to GRADE, the overall strength of the evidence supporting this outcome was moderate Table 4.

**Table 4:** Summary of the findings according to the GRADE guidelines for the FM + MP vs. no treatment comparison

Certainty assessment						№ of patients	Effect on FM + MP group	Certainty	Comment
№ of studies	Risk of bias	Inconsistency	Indirectness	Imprecision	Other considerations		Absolute (95% CI)		
1- Upper lip protrusion (assessed with mm)									
2	serious	not serious	not serious	not serious	none	64	2.52 (1.86, 3.18)	⨁⨁⨁◯ Moderate^a^	significant increase of 2.52 mm upper lip protrusion in patients treated with miniplate-anchored orthopedic facemask compared to the control group. (*P* < 0.00001)
2- Lower lip protrusion (assessed with mm)									
2	serious	not serious	not serious	not serious	none	64	-3.14 (-3.92, -2.36)	⨁⨁⨁◯ Moderate^a^	significant retraction of lower lip 3.14 mm in patients treated with FM + MP compared to the control group (*P* < 0.00001)
3- Soft tissue pogonion (assessed with mm)									
2	serious	not serious	not serious	not serious	none	64	-4.82 (-5.67, -3.98)	⨁⨁⨁◯ Moderate^a^	The chin moved backward significantly by 4.82 mm in the patients treated with FM + MP compared to control group. (*P* < 0.00001)

a. Downgrade one level for risk of bias

On the other hand, seven studies tested seven different appliances, including the orthodontic removable traction appliance (ORTA) [6], removable mandibular retractor (RMR) appliance [30], novel magnetic orthopaedic appliance (MOA-III) [34], FM associated with Bionator III (FM + Bio) [5], intermaxillary Class III elastics (C3E) anchored by a hybrid hyrax (HH) [27], reverse forsus [33], and miniscrew-anchored inverted forsus [26]. All of these studies showed a statistically significant improvement in upper lip protrusion in the treatment groups compared with the control groups. A summary of quantitative measurements in each study is described in Supplementary Table 5.

#### Lower lip protrusion

Fourteen studies reported lower lip protrusion changes compared to the untreated control groups [5, 6, 24–35]. Four studies [24, 29, 31, 33] compared the FM/RME and untreated control groups. Two studies [31, 33] used the vertical reference line (VRL) as a baseline, one study [29] measured the perpendicular distance between the lower lip’s anterior point and the subnasale-pogonion line, while another study [24] utilized the E line. Consequently, the standardized mean difference (SMD) was employed to combine these results effectively. The pooled estimate reported a statistically significant decrease of 0.84 mm lower lip protrusion in patients treated with FM/RME compared to the control group. (SMD = -0.84; 95% CI (-1.35, -0.34); *P* = 0.001; Fig. 4C). Heterogeneity was moderate (Chi^2^ = 6.49; *P* = 0.09; I^2^ = 54%). According to GRADE, the overall strength of the evidence supporting this outcome was moderate Table 2. However, when combining the studies that used the vertical reference line (VRL) as a baseline [31, 33], the pooled estimate showed a statistically significant decrease of 2.87 mm upper lip protrusion in patients treated with FM/RME compared to the control group. (MD = -2.87; 95% CI (-3.91, -1.83); *P* < 0.00001; Fig. 4D).

**Fig. 7:**
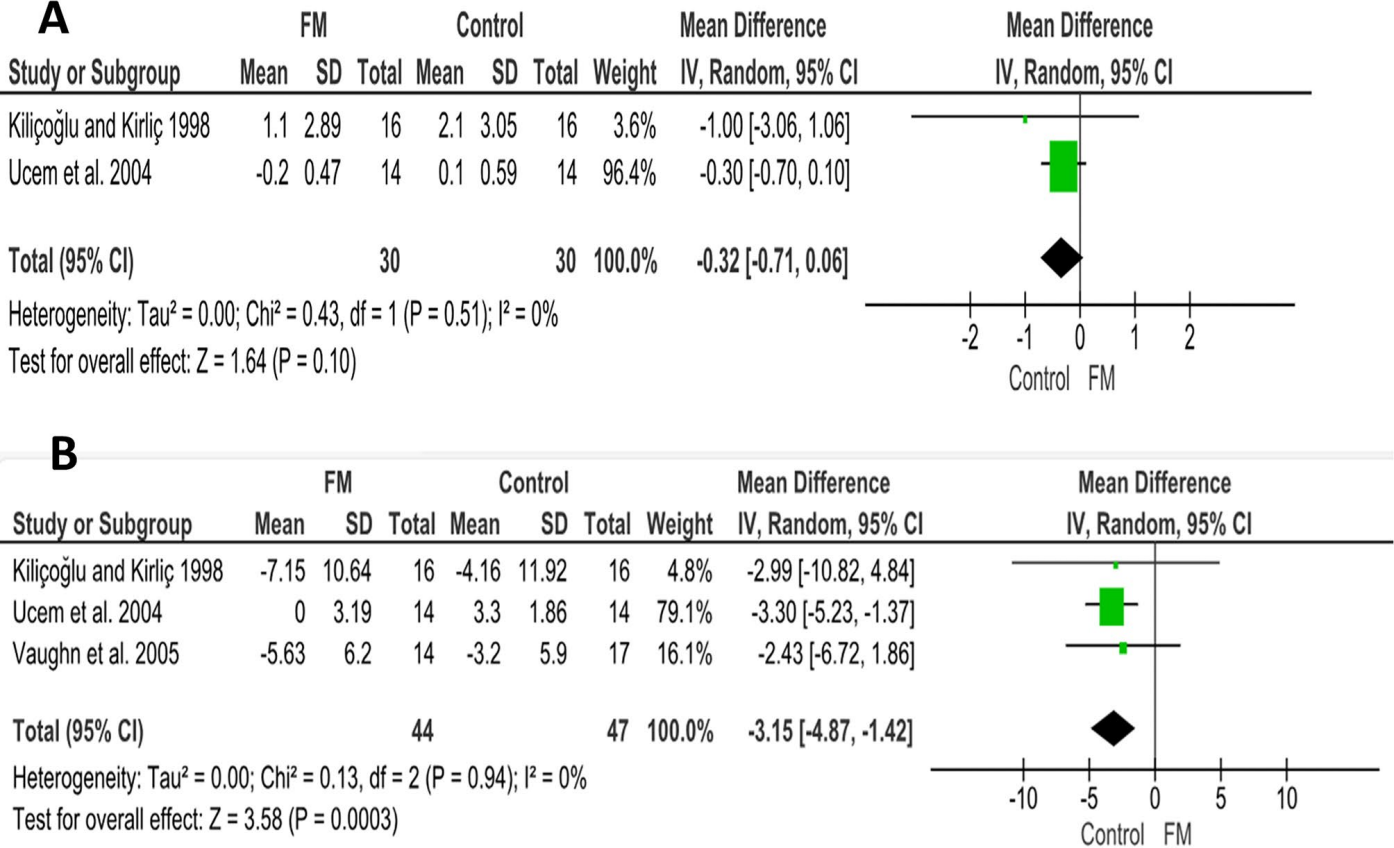
Forest plots of soft tissue changes associated with the FM appliance compared to the control group: (**A**) lower lip protrusion, (**B**) Nasolabial angle

Conversely, the results showed no statistical significance in lower lip protrusion between the FM and the untreated control groups. (MD = -0.32; 95% CI (-0.71, 0.06); *P* = 0.10; Fig. 7A). Heterogeneity was low (Chi^2^ = 0.43; *P* = 0.51; I^2^ = 0%). According to GRADE, the overall strength of the evidence supporting this outcome was low Table 5.

**Table 5:** Summary of the findings according to the GRADE guidelines for the FM vs. no treatment comparison

Certainty assessment						№ of patients	Effect on CA group	Certainty	Comment
№ of studies	Risk of bias	Inconsistency	Indirectness	Imprecision	Other considerations		Absolute (95% CI)		
1- Lower lip protrusion (assessed with mm)									
2	very serious	not serious	not serious	not serious	none	60	-0.32 (-0.71, 0.06)	⨁⨁◯◯ Low^a^	The difference was not statistically significant (*p* = 0.10)
2- Nasolabial angle (assessed with degrees)									
3	very serious	not serious	not serious	not serious	none	91	-3.15 (-4.87; -1.42)	⨁⨁◯◯ Low^b^	statistically significant decrease of 3.15 degrees in patients treated with FM compared to the control group. (*P* = 0.003)

a. Downgrade two levels for risk of bias

Akin et al. [24] and Alacrόn et al. [25] also reported changes in the lower lip protrusion after chincup treatment compared with the control group. The results showed a significant decrease of 2.63 mm lower lip protrusion in patients treated with CC compared to the control group. (MD = -2.63; 95% CI (-4.54, -0.72); *P* = 0.007; Fig. 5B). Heterogeneity was significant (Chi^2^ = 3.27; *P* = 0.07; I^2^ = 69%). According to GRADE, the overall strength of the evidence supporting this outcome was low Table 3.

When evaluating the lower lip changes associated FM + MP compared to a control group, the pooled estimate showed a significant retraction of the lower lip 3.14 mm in the treatment group. (MD = -3.14; 95% CI (-3.92, -2.36); *P* < 0.00001; Fig. 6B). Heterogeneity was low (Chi^2^ = 0.20; *P* = 0.65; I^2^ = 0%). According to GRADE, the overall strength of the evidence supporting this outcome was moderate Table 4.

In the same context, eight studies tested eight different appliances, including the orthodontic removable traction appliance (ORTA) [6], Removable mandibular retractor (RMR) appliance [30], novel magnetic orthopaedic appliance (MOA-III) [34], FM associated with Bionator III (FM + BIO) [5], Double-Plate Appliance (DPA) with 2 Class III elastics [35], intermaxillary Class III elastics (C3E) anchored by a hybrid hyrax (HH) [27], reverse forsus [33], and miniscrew-anchored inverted forsus [26]. Five of these studies showed a statistically significant retraction of the lower lip in the treatment groups compared with the control groups [26, 27, 30, 33, 34]. On the other hand, the remaining three studies [5, 6, 35] did not find any significant difference between the two groups. More details about quantitative measurements can be found in Supplementary Table 5.

#### Nasolabial angle

Nine included studies investigated the changes in the nasolabial angle when compared to an untreated control group [5, 6, 25, 26, 28–30, 35, 36]. Pavoni et al. [29] and Vaughn et al. [36] reported changes in the nasolabial angle after FM/RME treatment. The results showed a significant decrease of 4.73 degrees in patients treated with FM/RME compared to the control group. (MD = -4.73; 95% CI (-7.72, -1.73); *P* = 0.002; Fig. 4E). Heterogeneity was low (Chi^2^ = 0.01; *P* = 0.94; I^2^ = 0%). According to GRADE, the overall strength of the evidence supporting this outcome was moderate Table 2.

Three studies [28, 35, 36] focused on comparing between the FM group and the untreated control group. The pooled estimate showed a statistically significant decrease of 3.15 degrees in patients treated with FM compared to the control group. (MD = -3.15; 95% CI (-4.87, -1.42); *P* = 0.003; Fig. 7B). Heterogeneity was low (Chi^2^ = 0.13; *P* = 0.94; I^2^ = 0%). According to GRADE, the overall strength of the evidence supporting this outcome was low Table 5.

Five studies were conducted on five different appliances, including the ORTA appliance [6], the RMR appliance [30], the FM + Bio appliance [5], the DPA appliance with 2 Class III elastics [35], and miniscrew-anchored inverted forsus [26]. Only two of these studies [26, 30] reported a significant decrease in the nasolabial angle group in the treatment groups compared with the control groups, which evaluated miniscrew-anchored inverted forsus and RMR appliance, respectively. The remaining three studies did not find any difference between the two groups. Supplementary Table 5 contains more details about the quantitative measurements.

#### Profile changes

Six different trials [5, 25, 28–30, 34] were conducted to investigate the impact of early class III treatment using six different appliances on facial profile changes, including chincup [25], only FM [28], FM/RME [29], FM + BIO [5], MOA-III appliance [34], and the RMR appliance [30]. All of these studies showed a statistically significant improvement in Facial convexity angle in the treatment groups compared with the control groups. Supplementary Table 5 provides a summary of the quantitative measurements in each study.

#### Soft tissue pogonion (pg’)

Six included studies investigated the changes in soft tissue pogonion when compared to an untreated control group [25, 27, 28, 30–32]. The pooled estimate showed that the chin moved backward significantly by 4.82 mm in the patients treated with FM + MP compared to the control group. (MD = -4.82; 95% CI (-5.67, -3.98); *P* < 0.00001; Fig. 6C). Heterogeneity was low (Chi^2^ = 0.53; *P* = 0.46; I^2^ = 0%). According to GRADE, the overall strength of the evidence supporting this outcome was moderate Table 4.

Moreover, four separate trials [25, 27, 28, 30] examined the effects of early class III treatment with 4 different appliances on the changes in chin soft tissue. All these trials reported statistically significant retrusion in soft tissue pogonion in the treatment groups compared with the control groups.

#### Mentolabial angle

Three studies evaluated the effects of three different appliances on the mentolabial angle. The appliances evaluated were FM [28], the ORTA appliance [6], and the RMR appliance [30]. Only the study involving the RMR appliance showed a significant reduction in the mentolabial angle compared to the control group [30].

#### Upper lip thickness

Three trials [5, 25, 28] investigated the impact of three different appliances on upper lip thickness. These appliances included a chincup, only FM, and FM associated with Bionator III (FM + BIO). Among these studies, only the trial that employed FM + BIO revealed a statistically significant increase in upper lip thickness when compared to the control group [5].

#### Upper lip strain

In two separate trials [5, 28], the effects of two appliances on upper lip strain were studied. Cozza et al. observed a significant decrease in upper lip strain in the FM + BIO group compared to the control group. However, Kiliçoğlu and Kirliç’s study found no significant difference between the FM and control groups.

### Second: Appliance 1 vs. Appliance 2

This review includes thirteen studies that compare different orthopaedic appliances and their impact on soft tissue outcomes. For the meta-analysis, data on these outcomes was only considered when at least two studies used the same outcome measure for the same compared orthopaedic appliances. A summary of appliances compared in the review is provided in Table 6.

**Table 6:** Summary of appliances compared in the review

Comparisons	Outcomes	Number of studies	Effect size	*P*-value	Conclusion
FM/RME vs. untreated control	Upper lip protrusion (mm)	4	+ 1.58	*P* < 0.00001	Significant increase of 1.58 mm upper lip protrusion in patients treated with FM/RME compared to the control group.
	Lower lip protrusion (mm)	4	-0.84	*p* = 0.001	Significant decrease of 0.84 mm lower lip protrusion in patients treated with FM/RME
	Nasolabial angle (degrees)	2	-4.73	*P* = 0.002	Significant decrease of 4.73 degrees in patients treated with FM/RME compared to the control group
FM vs. untreated control	Upper lip protrusion (mm)	1	+ 2.6	*P* < 0.05	Significant increase of 2.6 mm upper lip protrusion in patients treated with FM compared to the control group.
	Lower lip protrusion (mm)	2	-0.32	*p* = 0.10	The difference was not statistically significant.
	Nasolabial angle (degrees)	3	-3.15	*P* = 0.003	statistically significant decrease of 3.15 degrees in patients treated with FM compared to the control group.
CC vs. untreated control	Upper lip protrusion (mm)	2	2.13	*P* < 0.00001	significant increase of 2.13 mm upper lip protrusion in patients treated with CC compared to the control group
	Lower lip protrusion (mm)	2	-2.63	*P* = 0.007	significant decrease of 2.63 mm lower lip protrusion in patients treated with CC compared to the control group
FM/MP vs. untreated control	Upper lip protrusion (mm)	2	2.52	*P* < 0.00001	significant increase of 2.52 mm upper lip protrusion in patients treated with miniplate-anchored orthopedic facemask compared to the control group
	Lower lip protrusion (mm)	2	-3.14	*P* < 0.00001	significant retraction of lower lip 3.14 mm in patients treated with FM + MP compared to the control group
	Soft tissue pogonion (mm)	2	-4.82	*P* < 0.00001	The chin moved backward significantly by 4.82 mm in the patients treated with FM + MP compared to control group
FM/RME vs. FM/MP	Upper lip protrusion (mm)	6	0.36	*P* = 0.32	Non-significant difference between the two groups
	Lower lip protrusion (mm)	5	0.19	*P* = 0.50	Non-significant difference between the two groups
	Nasolabial angle (degrees)	3	1.58	*P* = 0.04	significant decrease of 1.58 degree in patients treated with FM/RME compared to FM/MP group
	Soft tissue pogonion (mm)	3	0.33	*P* = 0.50	Non-significant difference between the two groups
FM/RME vs. Alt-RAMEC/FM	Upper lip protrusion (mm)	3	0.67	*P* = 0.02	significant changes with an increase in upper lip protrusion of 0.67 mm in patients treated with Alt-RAMEC/FM compared to FM/RME group
	Lower lip protrusion (mm)	3	0.01	*P* = 0.98	Non-significant difference between the two groups
	Nasolabial angle (degrees)	3	-0.27	*P* = 0.48	Non-significant difference between the two groups
	Soft tissue pogonion (mm)	2	0.08	*P* = 0.90	Non-significant difference between the two groups
FM/RME vs. Reverse Forsus	Upper lip protrusion (mm)	1	+ 0.88	*NS*	Non-significant difference between the two groups
	Lower lip protrusion (mm)	1	-0.08	*NS*	Non-significant difference between the two groups
Reverse Forsus vs. untreated control	Upper lip protrusion (mm)	1	1.32	*P* < 0.05	significant increase of 1.32 mm upper lip protrusion in patients treated with Reverse Forsus compared to the control group.
	Lower lip protrusion (mm)	1	-3.6	*P* < 0.01	significant decrease of 3.6 mm lower lip protrusion in patients treated with Reverse Forsus compared to the control group.
ORTA vs. untreated control	Upper lip protrusion (mm)	1	+ 1.77	*P* = 0.002	significant increase of 1.77 mm upper lip protrusion in patients treated with ORTA compared to the control group.
	Lower lip protrusion (mm)	1	-0.60	*P* = 0.166	Non-significant difference between the two groups
	Nasolabial angle (degrees)	1	0.505	*P* = 0.505	Non-significant difference between the two groups
	Mentolabial angle (degrees)	1	-5.24	P = 0.164	Non-significant difference between the two groups
RMR vs. untreated control	Upper lip protrusion (mm)	1	+ 4	*P* < 0.001	significant increase of 4 mm upper lip protrusion in patients treated with RMR compared to the control group.
	Lower lip protrusion (mm)	1	-1.21	*P* < 0.001	significant decrease of 1.21 mm lower lip protrusion in patients treated with RMR compared to the control group.
	Nasolabial angle (degrees)	1	-6.7	*P* < 0.001	significant decrease of 6.7 degrees in patients treated with RMR compared to the control group.
	Mentolabial angle (degrees)	1	+ 4.66	*P* < 0.001	significant increase of 4.66 degrees in patients treated with RMR compared to the control group.
	Profile facial angle (degrees)	1	+ 5.02	*P* < 0.001	significant increase of 5.02 degrees in patients treated with RMR compared to the control group.
CHS vs. FM	Lower lip protrusion (mm)	1	-0.39	*NS*	Non-significant difference between the two groups
	Nasolabial angle (degrees)	1	-0.9	*NS*	Non-significant difference between the two groups
miniscrew-anchored inverted FRD vs. untreated control.	Upper lip protrusion (mm)	1	0.83	*P* < 0.001	significant increase of 0.83 mm upper lip protrusion in patients treated with FRD compared to the control group.
	Lower lip protrusion (mm)	1	-1.71	*P* < 0.001	significant decrease of 1.71 mm lower lip protrusion in patients treated with FRD compared to the control group.
	Nasolabial angle (degrees)	1	-3.97	*P* < 0.001	significant decrease of 3.97 degrees in patients treated with FRD compared to the control group.
FM + BIO vs. untreated	Upper lip protrusion (mm)	1	+ 2.69	*P* < 0.001	significant increase of 2.69 mm upper lip protrusion in patients treated with FM + BIO compared to the control group.
	Lower lip protrusion (mm)	1	-0.54	*NS*	Non-significant difference between the two groups
	Nasolabial angle (degrees)	1	-1.04	*NS*	Non-significant difference between the two groups
	Profile facial angle (degrees)	1	3.77	*P* < 0.001	significant increase of 3.77 degrees in patients treated with FM + BIO compared to the control group.
	Upper lip thickness (mm)	1	1.15	*P* < 0.001	significant increase in upper lip thickness when compared to the control group
	Upper lip strain (mm)	1	-1.73	*P* < 0.001	significant decrease in upper lip strain in the FM + BIO group compared to the control group
(FM started after the completion of the Alt-RAMEC) vs. (FM started simultaneously with the Alt-RAMEC)	Upper lip protrusion (mm)		0.6	*NS*	Non-significant difference between the two groups
	Lower lip protrusion (mm)		-0.47	*NS*	Non-significant difference between the two groups
	Soft tissue pogonion (mm)		0.39	*NS*	Non-significant difference between the two groups
	Profile facial angle (degrees)		0.2	*NS*	Non-significant difference between the two groups
FM + RME vs. Mini maxillary protractor (MMP)	Upper lip protrusion (mm)	1	1.22	*P* = 0.03	significant increase of 1.22 mm upper lip protrusion in patients treated with MMP compared to FM/RME.
	Lower lip protrusion (mm)	1	-0.56	*NS*	Non-significant difference between the two groups
	Nasolabial angle (degrees)	1	0.32	*NS*	Non-significant difference between the two groups
	Profile facial angle (degrees)	1	0.16	*NS*	Non-significant difference between the two groups
Double-plate appliance (DPA) with 2 Class III elastics vs. FM	Lower lip protrusion (mm)	1	-1.2	*NS*	Non-significant difference between the two groups
	Nasolabial angle (degrees)	1	-0.18	*NS*	Non-significant difference between the two groups
EL + MP vs. FM + MP	Upper lip protrusion (mm)	1	0.06		Non-significant difference between the two groups
	Lower lip protrusion (mm)	1	0.05		Non-significant difference between the two groups
	Soft tissue pogonion (mm)	1	0.24		Non-significant difference between the two groups

#### Upper lip protrusion

**Fig. 8:**
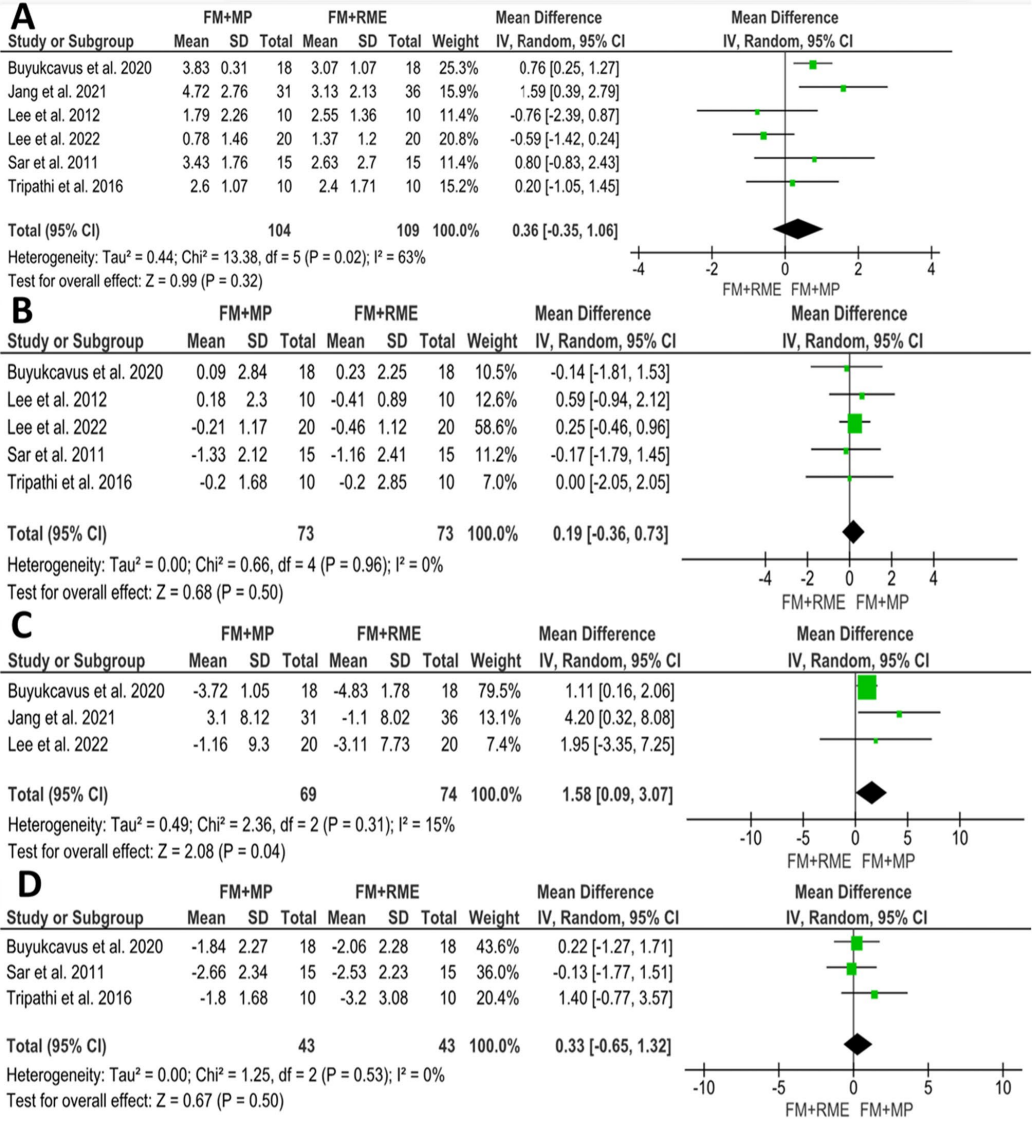
Forest plots of soft tissue changes associated with FM/MP vs. FM/RME treatment: **A**) Upper lip protrusion, **B**) Lower lip protrusion, **C**) Nasolabial angle, **D**) Soft tissue pogonion (Pg’)

Figure 8A present graphical depictions of the upper lip changes caused by FM/MP vs. FM/RME treatment. The pooled estimate showed a non-significant difference between the two groups. (MD = 0.36; 95% CI (-0.35, 1.06); *P* = 0.32). Heterogeneity was significant (Chi^2^ = 13.38; *P* = 0.02; I^2^ = 63%). According to GRADE, the overall strength of the evidence supporting this outcome was low Table 7.

**Table 7:** Summary of the findings according to the GRADE guidelines for the FM/RME vs. FM/MP comparison

Certainty assessment						№ of patients	Effect on FM/MP group	Certainty	Comment
№ of studies	Risk of bias	Inconsistency	Indirectness	Imprecision	Other considerations		Absolute (95% CI)		
1- Upper lip protrusion (assessed with mm)									
6	serious	serious	not serious	not serious	none	213	0.36 (-0.35, 1.06)	⨁⨁◯◯ Low^b^	Non-significant difference between the two groups. (*P* = 0.32)
2- Lower lip protrusion (assessed with mm)									
5	serious	not serious	not serious	not serious	none	146	0.19 (-0.36, 0.73)	⨁⨁⨁◯ Moderate^a^	Non-significant difference between the two groups. (*P* = 0.50)
3- Nasolabial angle (assessed with degrees)									
3	serious	not serious	not serious	not serious	none	143	1.58 (0.09, 3.07)	⨁⨁⨁◯ Moderate^a^	significant decrease of 1.58 degrees in patients treated with FM/RME compared to the FM/MP group. (*P* = 0.04)
4- Soft tissue Pogonion (assessed with mm)									
3	serious	not serious	not serious	not serious	none	86	0.33 (-0.65, 1.32)	⨁⨁⨁◯ Moderate^a^	Non-significant difference between the two groups. (*P* = 0.50)

a. Downgrade one level for risk of bias

b. Downgrade one level for risk of bias, and one level due to inconsistency

**Fig. 9:**
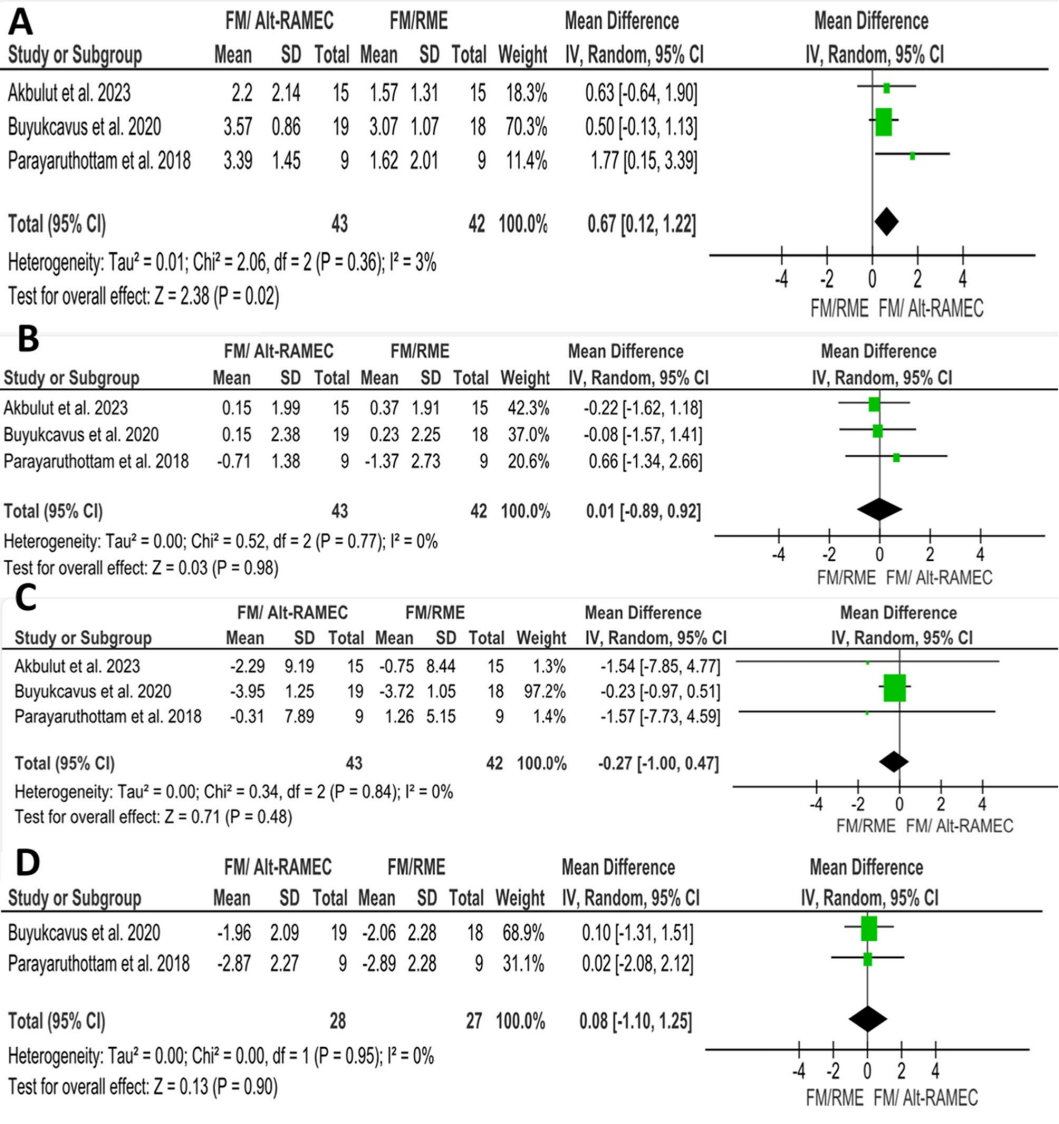
Forest plots of soft tissue changes associated with Alt-RAMEC/FM vs. FM/RME treatment: **A**) Upper lip protrusion, **B**) Lower lip protrusion, **C**) Nasolabial angle, **D**) Soft tissue pogonion (Pg’)

Additionally, Akbulut et al. [37]; Buyukcavus et al. [38] and Parayaruthottam et al. [39], compared the changes in upper lip protrusion using Alt-RAMEC/FM versus FM/RME. The pooled estimate showed significant changes with an increase in upper lip protrusion of 0.67 mm in patients treated with the Alt-RAMEC/FM compared to the FM/RME group. (MD = 0.67; 95% CI (0.12, 1.22); *P* = 0.02; Fig. 9A). Heterogeneity was low (Chi^2^ = 2.06; *P* = 0.36; I^2^ = 3%). According to GRADE, the overall strength of the evidence supporting this outcome was moderate Table 8.

**Table 8:** Summary of the findings according to the GRADE guidelines for the FM/RME vs. Alt-RAMEC/FM comparison

Certainty assessment						№ of patients	Effect on Alt-RAMEC/FM group	Certainty	Comment	
№ of studies	Risk of bias	Inconsistency	Indirectness	Imprecision	Other considerations		Absolute (95% CI)			
1- Upper lip protrusion (assessed with mm)										
3	serious	not serious	not serious	not serious	none	85	0.67 (0.12, 1.22)	⨁⨁⨁◯ Moderate^a^	significant changes with an increase in upper lip protrusion of 0.67 mm in patients treated with Alt-RAMEC/FM compared to the FM/RME group (*P* = 0.02)	
2- Lower lip protrusion (assessed with mm)										
3	serious	not serious	not serious	not serious	none	85	0.01 (-0.89, 0.92)	⨁⨁⨁◯ Moderate^a^	Non-significant difference between the two groups. (*P* = 0.98)	
3- Nasolabial angle (assessed with degrees)										
3	serious	not serious	not serious	not serious	none	85	-0.27 (-1.00, 0.47)	⨁⨁⨁◯ Moderate^a^	Non-significant difference between the two groups. (*P* = 0.48)	
4- Soft tissue pogonion (assessed with mm)										
2	serious	not serious	not serious	not serious	none	55	0.08 (-1.1, 1.25)	⨁⨁⨁◯ Moderate^a^	Non-significant difference between the two groups. (*P* = 0.90)	

a. Downgrade one level for risk of bias

Akin et al. [24] investigated the effect of chincup versus facemask on upper lip protrusion and they found no statistical difference between the two groups. Similarly, Liu et al. [40] compared banded versus modified appliances for anchorage during facemask maxillary protraction. They asserted that no statistically significant difference was detected between the two groups. Yavan et al. [33] also found no significant difference between the Reverse Forsus appliance and the FM/RPE in their three-arm design of the RCT consisting of 15 patients for each group. Canturk and Celikoglu [41] tested the soft tissue changes induced by facemask (FM) started simultaneously and after an alternate rapid maxillary expansion and constriction (Alt-RAMEC) procedure. They highlighted the equal mean difference in upper lip protrusion for both groups. Ağlarcı et al. [42] compare the upper lip changes between the facemask and skeletal anchorage therapy with intermaxillary elastics in patients with maxillary retrognathia. The RCT administered by Sar et al. [32] indicated no significant difference between the MP + FM group and the MP + CIII elastics group.

Moreover, Celikoglu et al. [43] found that the upper lip moved forward 1.22 mm more in the mini maxillary protractor (MMP) group compared to the FM/RME group at a *p*-value of less than 0.05 in their two-arm RCT, with 16 patients included in each group. James et al. [44] showed that the Alt-RAMEC /FM/Class III elastics group resulted in a greater increase in upper lip protrusion than the Alt-RAMEC/RH group with a statistically significant difference between the groups (*P* = 0.002).

#### Lower lip protrusion

Five studies [8, 31, 38, 45, 46] involving 146 patients (73 FM/MP, 73 FM/RME) assessed the changes in the lower lip protrusion with the FM/MP versus the FM/RME. The pooled estimate indicates no significant difference between the two groups. (MD = 0.19; 95% CI (-0.36, 0.73); *P* = 0.50; Fig. 8B). Heterogeneity was low (Chi^2^ = 0.66; *P* = 0.96; I^2^ = 0%). According to GRADE, the overall strength of the evidence supporting this outcome was moderate Table 7.

Comparing Alt-RAMEC/FM to FM/RME, three studies were pooled to evaluate an estimate of the combined effect size of the changes in lower lip protrusion. The results showed a non-significant difference between the two groups. (MD = 0.01; 95% CI (-0.89, 0.92); *P* = 0.98; Fig. 9B). Heterogeneity was low (Chi^2^ = 0.52; *P* = 0.77; I^2^ = 0%). According to GRADE, the overall strength of the evidence supporting this outcome was moderate Table 8.

Several studies have compared the effectiveness of different orthodontic appliances on the protrusion of the lower lip. However, the results of these studies have shown no significant difference between the groups. For instance, Akin et al. [24] compared chincup versus facemask, Liu et al. [40] compared banded versus modified appliances, and Yavan et al. [33] compared the Reverse Forsus appliance and the FM/RPE appliance. Similarly, Lim et al. [47] evaluated the creative horseshoe appliance’s (CHS) effectiveness versus a conventional facemask appliance and found no significant difference between the two groups. Other studies conducted by Canturk and Celikoglu [41], James et al. [44], Sar et al. [32], Ucem et al. [35], and Ağlarcı et al. [42] also showed no significant difference between different orthodontic appliances.

#### Nasolabial angle

Three studies [38, 46, 48] focused on comparing FM/MP and FM/RME. The pooled estimate showed a statistically significant decrease of 1.58 degrees in patients treated with the FM/RME compared to the FM/MP group. (MD = 1.58; 95% CI (0.09, 3.07); *P* = 0.04; Fig. 8C). Heterogeneity was low (Chi^2^ = 2.36; *P* = 0.31; I^2^ = 15%). According to GRADE, the overall strength of the evidence supporting this outcome was moderate.

Comparing Alt-RAMEC/FM to FM/RME, three studies [37–39] were pooled to evaluate an estimate of the combined effect size of the changes in the nasolabial angle. The results showed a non-significant difference between the two groups. (MD = -0.27; 95% CI (-1.00, 0.47); *P* = 0.48; Fig. 9C). Heterogeneity was low (Chi^2^ = 0.34; *P* = 0.84; I^2^ = 0%). According to GRADE, the overall strength of the evidence supporting this outcome was moderate Table 8.

In the same context, Vaughn et al. [33] found no significant difference in the nasolabial angle between the FM and the FM/RME treatment. Ucem et al. [35] also reported no significant difference in nasolabial angle changes between the Facemask and the DPA with 2 Class III elastics. Similarly, Lim et al. [47] found no statistically significant difference between the two groups in the nasolabial angle (*p* = 0.43). Celikoglu et al. [43] found no difference between the MMP group and the FM/RME group at a *p*-value of 0.22. Ağlarcı et al. [42] reported that the difference between the groups was insignificant (*p* = 0.245). Finally, James et al. [44] demonstrated that the use of Alt-RAMEC/FM/Class III elastics significantly decreases the nasolabial angle compared to the Alt-RAMEC/RH group (*P* = 0.001).

#### Profile changes

Only two studies have compared the effect of different appliances on facial profile changes [41, 43]. A trial by Canturk and Celikoglu [41] investigated the soft tissue changes induced by a facemask started simultaneously and after an alternate rapid maxillary expansion and constriction (Alt-RAMEC) procedure. They reported no significant difference in the convexity angle changes between the two groups (*p* = 0.85). In another two-arm RCT study by Celikoglu et al. [42], with 16 patients in each group, no significant difference was found in profile changes between the mini maxillary protractor (MMP) group and the FM/RME group (*p* = 0.87).

#### Soft tissue pogonion (pg’)

Three articles [8, 31, 38] involving 86 patients (43 FM/MP, 43 FM/RME) assessed the changes in soft tissue pogonion with the FM/MP versus the FM/RME. Data synthesis revealed that there was a non-significant difference between the two groups. (MD = 0.33; 95% CI (-0.65, 1.32); *P* = 0.50; Fig. 8D). Heterogeneity was low (Chi^2^ = 1.25; *P* = 0.53; I^2^ = 0%). According to GRADE, the overall strength of the evidence supporting this outcome was moderate Table 7.

Comparing Alt-RAMEC/FM to FM/RME, two studies [38, 39] were pooled to evaluate an estimate of the combined effect size of the changes in soft tissue pogonion. The results showed a non-significant difference between the two groups. (MD = 0.08; 95% CI -1.1, 1.25); *P* = 0.90 Fig. 9D). Heterogeneity was low (Chi^2^ = 0.0; *P* = 0.95; I^2^ = 0%). According to GRADE, the overall strength of the evidence supporting this outcome was moderate Table 8.

#### Assessment method

all studies included in this review used 2D cephalometric imaging, with the exception of only two studies that employed 3D imaging. The first study conducted by Ozbailen [49] examined soft tissue changes resulting from maxillary protraction employing RME or the ALT-RAMEC protocol via three-dimensional (3D) stereophotogrammetry. The analysis revealed a noticeable increase in upper lip protrusion in both the FM/RME and Alt-RAMEC/FM groups in comparison to the control group, with reported mean differences of 1.06 ± 0.48 mm and 1.22 ± 0.64 mm, respectively. Furthermore, a statistically significant backward was observed for the lower lip and the chin in both the FM/RME and Alt-RAMEC/FM groups when compared to the control group, with mean differences of − 1.06 ± 1.26 mm and − 0.68 ± 0.45 mm, respectively. Conversely, no significant disparities were identified between the RPE/FM and Alt-RAMEC/FM groups.

The second study evaluated the soft tissue effects of the Alt-RAMEC/FM protocol using Three-dimensional photographs superimposed by 3dMD patient software and then transferred to MIMICs software. The researchers observed a significant anterior movement in all measured points during the treatment. Moreover, there were noteworthy increases in nasal width, specifically in the subalare r–l (1.71 ± 2.34 mm) and alar curvature r–l (1.96 ± 1.4 mm) distances. Conversely, within the control group, only the b point (1.03 ± 1.84 mm) exhibited significant anterior movement attributed to natural growth [50].

## Discussion

Achieving a harmonious soft tissue profile and enhancing the facial appearance represent significant goals in the early treatment of class III growing patients. Consequently, the accurate evaluation of soft tissue becomes a crucial aspect of this type of orthodontic treatment. This review aims to summarize the current evidence about the effects of Class III orthopaedic treatment on the facial soft tissues.

The timing of Class III treatment is a significant consideration due to the multifactorial nature of its etiology and the complexities associated with its management [51]. Patients presenting with Class III malocclusion require prolonged monitoring, often extending into young adulthood, as a result of potentially adverse growth patterns [52]. Some studies indicate that the most favorable periods for intervention occur during the early developmental phases (before the age of 7) [53, 54]. Conversely, other studies suggest that the most appropriate timeframe for intervention is during late prepubertal phases (after 9 years of age) [55, 56]. Latest current research shows that there were no significant differences when treating Class III patients, either during an early phase (< 7 years of age) or during a prepubertal phase (> 9 years of age) [51–55].

This review focuses on growing patients with skeletal Class III malocclusion within the age range of 7 to 12 years, which aligns with recent studies that have explored the skeletal and dentoalveolar changes associated with Class III treatment [12]. Additionally, several studies highlight the importance of starting treatment after the eruption of the incisors. Evidence suggests that the best time to intervene in cases of Class III malocclusion is when the maxillary incisors have erupted. This timing can promote a favorable overjet and overbite, which ultimately helps maintain proper anterior occlusion by the end of the treatment [56].

According to this systematic review, application of FM/RME protocol resulted in an increase of 1.58 mm in upper lip protrusion and a decrease in the nasolabial angle by 4.73 degrees in the treatment group, compared to the control group. This favourable outcome was probably related to the anterior growth of the maxilla and protrusion of the upper incisors. This treatment was also associated with a decrease in lower lip protrusion of 0.84 mm, which could be attributed to the clockwise rotation of the mandible and retrusion of the lower incisors. Only one study has examined the facial profile changes associated with FM/RME, and it found a significant reduction of 4.4 degrees in the profile facial angle in the FM/RME group compared to the control group [29]. This result can be related to the long-term improvement of the intermaxillary sagittal skeletal relationship associated with a significant reduction in mandibular protrusion, as several studies have described [57, 58]. The observed soft tissue effects appeared to result from the induced skeletal changes. This is consistent with observations by Yavuz et al. [59] that significant correlations were found between changes in the sagittal relationships of skeletal and soft tissue profiles in both the maxilla and the mandible. The chincup treatment resulted in an increase of 2.13 mm in upper lip protrusion and a decrease of 2.63 mm in lower lip protrusion compared to controls. These findings, which demonstrate consistency with previous research [24, 60], may suggest a direct impact of the chincup on the skeletal structure of the mandible, particularly with regard to mandibular retrusion subsequent to the treatment regimen.

Moving to skeletal anchorage Class III appliances, numerous authors have investigated the application of this technique for orthopaedic treatment. A systematic review conducted by Rodríguez de Guzmán-Barrera et al. [61] pinpointed that using miniplates and miniscrews offers several advantages. These include fewer unwanted dental effects compared to dental anchorage [32, 62–64], greater skeletal effect [32, 63], enhanced maxillary advancement [64, 65], improved overjet and molar relationships [66], and reduced clockwise rotation of the mandible [64]. However, it is important to note that skeletal anchorage has certain drawbacks. The procedures involved are invasive, necessitating surgical intervention for both the insertion and removal [63, 67]. Additionally, some components may lack stability throughout the treatment [66]. Reported side effects include postoperative inflammation, irritation of the tissues adjacent to the miniscrews, and the accumulation of food particles in the area [62].

Regarding the indication for the use of this technique compared to the traditional technique, there is no consensus between studies [61]. However, the general recommendation is to start maxillary traction using a traditional facemask during the early mixed dentition stage, which typically occurs around 8 years of age. This timing is crucial to achieve maximum skeletal effects, as the responsiveness of the sutures to treatment decreases with age. In contrast, when using skeletal anchorage, treatment generally starts at a later age, around 10 years old. This later initiation is advantageous because the characteristics of the bone at this age facilitate proper placement of the anchorage and enhance its stability [32, 64, 66]. This aligns with the findings of the studies included in this review, all of which involved patients older than 10 years.

According to this review, the pooled estimate showed a significant increase of 1.82 mm upper lip protrusion, 3.14 mm significant retrusion of the lower lip, and a backward movement of the chin by 4.8 mm in patients treated with miniplate-anchored orthopaedic facemask compared to the control group. These outcomes are predictable as soft tissues typically follow the underlying hard tissues [8, 32]. According to this review, there was no difference between the FM/RME and the FM/MP protocols regarding the protrusion of the upper lip, lower lip, and soft tissue pogonion but a significant decrease in the nasolabial angle was observed in the FM/RME compared to the FM/MP. The reason behind this difference could be attributed to the fact that the RME pushes the anterior teeth forward, mesial dental movement and labial tipping of the maxillary incisors occur with the FM/RME. Conversely, the FM/MP moves the maxilla forward, which tends to keep the maxillary incisors upright due to the increased lip pressure [48]. These findings are consistent with observations by Eissa et al. [23] who evaluated the soft tissue effects of an inverted forsus appliance anchored with miniscrews compared to a control group. The treated group exhibited significant retrusion of the lower lip, which was evidenced by changes in the lower lip to the E plane relationship. Additionally, the treated group showed significant upper lip protrusion, which could be attributed to forward maxillary growth and mesial movement of maxillary teeth. This could be the main reason for the significant reduction in the nasolabial angle.

Within the same context, Kamel et al. [24] evaluated the effects of MAMP in growing patients with Class III malocclusion. The authors observed significant forward movement of the upper lip approximately 2.9 mm compared to the untreated group. The lower lip and soft tissue pogonion were restrained in the treated group in comparison with significant protrusions in the control group. These results could be attributed to the significant restriction of the mandibular growth with the negligible backward rotation of the mandible.

In exploring the differences in treatment protocols, studies utilizing non-skeletal traction adhered to a conventional approach. Extraoral elastics were secured to hooks positioned in the canine region on the buccal sides of the expanders, with a force applied ranging from 400 to 500 g per side, at an angle of approximately 30° below the occlusal plane [24, 29, 31, 33]. Conversely, studies employing skeletal anchorage exhibited variability in their protocols. They utilized two miniplates placed on the lateral nasal wall of the maxilla and applied a force of 400 g on each side [8, 31, 45, 46, 48]. In another study, Şar et al. [32] used this protocol for one group, while in a second group, they positioned two miniplates on the symphysis, using intermaxillary elastic bands connected to a maxillary expander fixed to the upper arch. After one week, they escalated the force to 500 g, which was maintained continuously for a full 24 h. It is important to note that there are no studies examining the soft tissue changes of the bollard miniplates technique, which is recognized as an effective option for treating skeletal Class III malocclusion, particularly in older adolescents. A study by De Clerck and Swennen [66] found that skeletal anchorage using bollard miniplates is effective for bone-anchored maxillary protraction (BAMP). Therefore, we recommend that future research should focus on evaluating the effectiveness of this technique, as well as any associated changes in soft tissue.

Another efficacious technique employed to address Class III malocclusion is alternate rapid maxillary expansion and constriction (Alt-RAMEC). This modality involves mobilizing the circummaxillary sutures through repetitive opening and closing of the expansion screw, usually for seven to nine weeks [68, 69]. This process leads to a weakening of the sutures which, in turn, facilitates the maxillary forward movement [37]. Comparing Alt-RAMEC/FM to FM/RME, this review did not find any difference in soft tissue outcomes, except for the upper lip protrusion. The Alt-RAMEC group exhibited a more pronounced anterior movement of the upper lip by 0.67 mm compared to the RME group. This could be due to the significant maxillary skeletal and dental changes in the Alt-RAMEC group such as the significant forward movement of the maxillary apical base (SNA) and the proclination of the maxillary incisors [39]. These findings are similar to those of previous studies, suggesting that the Alt-RAMEC protocol, when used alongside a facemask, is more effective than conventional RME in bringing about the protraction of the retruded maxilla [70–72]. Isci et al. [72], Masucci et al. [71] and Wilmes et al. [70] concluded that the Alt-RAMEC/FM protocol resulted in a more effective advancement of the maxilla and greater intermaxillary changes compared to the FM/RME protocol in developing Class III patients.

The review included several comparisons between different appliances, which were analyzed in individual articles and could not be combined into a single analysis. For example, comparisons were made between the creative horseshoe appliance (CHS) versus a conventional facemask appliance [47], banded versus modified appliances [40], Reverse Forsus appliance versus FM/RPE [33], and FM started simultaneously and after Alt-RAMEC [41]. None of these comparisons found significant differences concerning facial soft tissues, except for some specific outcomes that will be mentioned in the subsequent paragraph. This lack of significant differences may be due to the similar effects of the compared appliances on soft tissues, despite their different designs.

Moreover, Celikoglu et al. [43] found that the upper lip moved forward 1.22 mm more in the mini maxillary protractor (MMP) group compared to the FM/RME group. The observed changes were attributed to the greater anterior movement of the maxilla and the maxillary incisors in the MMP group compared to the FM/RME group. Furthermore, the MMP group likely experienced more pronounced effects on the upper lip. which was in agreement with the findings of James et al. [44] that showed that the Alt-RAMEC /FM/Class III elastics group resulted in a greater increase in upper lip protrusion compared to the Alt-RAMEC /RH group.

Concerning the mentolabial angle, three different studies evaluated this outcome using three different appliances: FM [28], ORTA appliance [6], and the RMR appliance [30]. Only the study involving the RMR appliance demonstrated a significant reduction in the mentolabial angle compared to the control group [30]. This could be due to the smaller anterior movement of the lower lip in the treated group, which may be a result of a considerable decrease in mandibular dentoalveolar protrusion and a significant reduction in total mandibular length, ultimately leading to a decreased mentolabial angle.

Regarding lip thickness and lip strain, only one trial showed a statistically significant difference in upper lip thickness and strain [5]. The FM + Bio appliance demonstrated a significant decrease in upper lip strain and a significant increase in upper lip thickness compared to the untreated control group. These findings can be attributed to dentoalveolar changes that resulted in differing correlations between the dentoalveolar and soft-tissue changes in the FM + BIO group compared to the control group. Specifically, in the FM + BIO group, an increased overjet and upper-incisor proclination were associated with the advancement of the upper lip, decreased upper lip strain, and increased upper lip thickness.

The findings in this review were based on studies that exclusively utilized 2D cephalometric imaging, with only two studies using 3D imaging [49, 50]. It’s important to note that the results drawn from these studies were based on a two-dimensional analysis of cephalometric images, which may not provide complete or accurate information due to the limitations of 2D imaging in capturing the complex three-dimensional structures of the craniofacial region. Hence, there is a crucial need for more randomized controlled trials that utilize comprehensive 3D analysis to corroborate and validate and support these findings.

## Conclusions

Based on the currently available data, it can be concluded that early orthopedic treatment in skeletal Class III patients leads to positive effects on facial soft tissues. The facemask with rapid maxillary expansion (FM/RME) protocol demonstrated improvements in upper lip protrusion, nasolabial angle, and lower lip protrusion compared to untreated patients. Additionally, the use of a miniplate-anchored orthopedic facemask (FM/MP) was associated with significant enhancements in upper and lower lip protrusion, as well as chin protrusion compared to the untreated control group. The only notable difference between FM/RME and FM/MP was a reduction in the nasolabial angle in the FM/RME group. No differences in soft tissue outcomes were observed between the alternate rapid maxillary expansion and constriction with facemask (Alt-RAMEC/FM) and FM/RME protocols, except for a more pronounced anterior movement of the upper lip in the Alt-RAMEC group. According to the GRADE framework, the overall strength of the evidence supporting these findings ranged from low to moderate.

### Limitations

The primary limitation of this review is the lack of high-quality 3D analyses, highlighting the need for additional studies with rigorous methodologies that focus specifically on study design and precise facial soft tissue quantification. Furthermore, the limited number of studies that have investigated certain appliances represents a limitation of this review, as numerous forest plots were generated based on the synthesis of data from only two studies. Another limitation stems from the fact that some appliances have only been evaluated in isolated studies, which limits the ability to gather sufficient evidence regarding their impacts on soft tissues.

## Electronic supplementary material

Below is the link to the electronic supplementary material.

Supplementary Material 1

Supplementary Material 2

Supplementary Material 3

Supplementary Material 4

Supplementary Material 5

## Data Availability

No datasets were generated or analysed during the current study.
