# Supplementary material for: Soft tissue changes associated with Class III orthopaedic treatment in growing patients: a systematic review and meta-analysis
Source: Prog Orthod. 2025 Mar 17;26:10. doi: 10.1186/s40510-025-00558-2 (PMC11911289; doi:10.1186/s40510-025-00558-2)
Supplement: Supplementary file 1 — Supplementary Material 1 [file 40510_2025_558_MOESM1_ESM.docx]

| **Supplementary** **Table 1: Electronic Search Strategy** | |  |
| --- | --- | --- |
| **Database** | **Search Strategy** | **Results** |
| **CENTRAL (The Cochrane Library)**  From inception up to 29-7-2024 Search field:  All text | #1 (“Skeletal Class iii” OR “Class iii Malocclusion” OR “maxillary retrusion” OR “mandibular protrusion” OR “mandibular hyperplasia” OR “maxillary hypoplasia” OR “anterior crossbite” OR “reverse occlusion”)  #2 (Functional OR “extraoral” OR “extra-oral” OR “facemask” OR “face mask” OR “chin cup” OR “reverse headgear” OR “intermaxillary traction”)  #3 (face OR facial OR mouth OR nose OR nasal OR naso* OR lip OR labial OR profile OR soft tissue)  #4 #1 AND #2 AND #3 | #4=76 |
| **EMBASE via OVID**  From inception up to 29-7-2024 | #1 (“Skeletal Class iii” OR “Class iii Malocclusion” OR “maxillary retrusion” OR “mandibular protrusion” OR “mandibular hyperplasia” OR “maxillary hypoplasia” OR “anterior crossbite” OR “reverse occlusion”)  #2 (Functional OR “extraoral” OR “extra-oral” OR “facemask” OR “face mask” OR “chin cup” OR “reverse headgear” OR “intermaxillary traction”)  #3 (face OR facial OR mouth OR nose OR nasal OR naso* OR lip OR labial OR profile OR soft tissue)  #4 #1 AND #2 AND #3 | #4=559 |
| **PubMed**  From inception up to 29-7-2024 Search Builder:  All fields | #1 (“Skeletal Class iii” OR “Class iii Malocclusion” OR “maxillary retrusion” OR “mandibular protrusion” OR “mandibular hyperplasia” OR “maxillary hypoplasia” OR “anterior crossbite” OR “reverse occlusion”)  #2 (Functional OR “extraoral” OR “extra-oral” OR “facemask” OR “face mask” OR “chin cup” OR “reverse headgear” OR “intermaxillary traction”)  #3 (face OR facial OR mouth OR nose OR nasal OR naso* OR lip OR labial OR profile OR soft tissue)  #4 #1 AND #2 AND #3 | #4=1708 |
| **Scopus**  From inception up to 29-7-2024 | #1 TITLE-ABS-KEY (“Skeletal Class iii” OR “Class iii Malocclusion” OR “maxillary retrusion” OR “mandibular protrusion” OR “mandibular hyperplasia” OR “maxillary hypoplasia” OR “anterior crossbite” OR “reverse occlusion”)  #2 TITLE-ABS-KEY (Functional OR “extraoral” OR “extra-oral” OR “facemask” OR “face mask” OR “chin cup” OR “reverse headgear” OR “intermaxillary traction”)  #3 TITLE-ABS-KEY (face OR facial OR mouth OR nose OR nasal OR naso* OR lip OR labial OR profile OR soft tissue)  #4 #1 AND #2 AND #3 | #4=147 |
| **Web of Science ™**  All databases  From inception up to 29-7-2024 | #1 (“Skeletal Class iii” OR “Class iii Malocclusion” OR “maxillary retrusion” OR “mandibular protrusion” OR “mandibular hyperplasia” OR “maxillary hypoplasia” OR “anterior crossbite” OR “reverse occlusion”)  #2 (Functional OR “extraoral” OR “extra-oral” OR “facemask” OR “face mask” OR “chin cup” OR “reverse headgear” OR “intermaxillary traction”)  #3 (face OR facial OR mouth OR nose OR nasal OR naso* OR lip OR labial OR profile OR soft tissue)  #4 #1 AND #2 AND #3 | #4=575 |
| **Trip**  From inception up to 29-7-2024 | ("skeletal class iii" OR "class iii malocclusion" OR "maxillary retrusion" OR "mandibular protrusion" OR "mandibular hyperplasia" OR "maxillary hypoplasia" OR "anterior crossbite" OR "reverse occlusion") AND (functional OR "extraoral" OR "extra-oral" OR "facemask" OR "face mask" OR "chin cup" OR "reverse headgear" OR "intermaxillary traction") AND (face OR facial OR mouth OR nose OR nasal OR naso* OR lip OR labial OR profile OR soft tissue) | 239 |
| **ProQuest**  From inception up to 29-7-2024 | ab(“Skeletal Class iii” OR “Class iii Malocclusion” OR “maxillary retrusion” OR “mandibular protrusion” OR “mandibular hyperplasia” OR “maxillary hypoplasia” OR “anterior crossbite” OR “reverse occlusion”) AND ab(Functional OR “extraoral” OR “extra-oral” OR “facemask” OR “face mask” OR “chin cup” OR “reverse headgear” OR “intermaxillary traction”) AND ab(face OR facial OR mouth OR nose OR nasal OR naso* OR lip OR labial OR profile OR soft tissue) | 413 |
| **ClinicalTrials.gov**  29-7-2024 | ("skeletal class iii" OR "class iii malocclusion" OR "maxillary retrusion" OR "mandibular protrusion" OR "mandibular hyperplasia" OR "maxillary hypoplasia" OR "anterior crossbite" OR "reverse occlusion") AND (functional OR "extraoral" OR "extra-oral" OR "facemask" OR "face mask" OR "chin cup" OR "reverse headgear" OR "intermaxillary traction") | 17 |
| **World Health Organization International Clinical Trials Registry Platform (ICTRP)**  **Search Portal**  **Advanced search (Title)**  29-7-2024 | ("skeletal class iii" OR "class iii malocclusion" OR "maxillary retrusion" OR "mandibular protrusion" OR "mandibular hyperplasia" OR "maxillary hypoplasia" OR "anterior crossbite" OR "reverse occlusion") AND (functional OR "extraoral" OR "extra-oral" OR "facemask" OR "face mask" OR "chin cup" OR "reverse headgear" OR "intermaxillary traction") | 21 |
