# Supplementary material for: Soft tissue changes associated with Class III orthopaedic treatment in growing patients: a systematic review and meta-analysis
Source: Prog Orthod. 2025 Mar 17;26:10. doi: 10.1186/s40510-025-00558-2 (PMC11911289; doi:10.1186/s40510-025-00558-2)
Supplement: Supplementary file 2 — Supplementary Material 2 [file 40510_2025_558_MOESM2_ESM.docx]

Supplementary Table 2. Excluded articles after full-text assessment.

|  | Article | Reason |
| --- | --- | --- |
|  | Kurt H, Alioğlu C, Karayazgan B, Tuncer N, Kılıçoğlu H. The effects of two methods of Class III malocclusion treatment on temporomandibular disorders. Eur J Orthod. 2011 Dec;33(6):636-41. doi: 10.1093/ejo/cjq114. Epub 2010 Dec 27. PMID: 21187525. | No eligible outcome assessed |
|  | J. Seehra, P. S. Fleming, N. Mandall, A. T. DiBiase; A comparison of two different techniques for early correction of Class III malocclusion. Angle Orthod 1 January 2012; 82 (1): 96–101. doi: <https://doi.org/10.2319/032011-197.1> | No eligible outcome assessed |
|  | Masucci C, Franchi L, Defraia E, Mucedero M, Cozza P, Baccetti T. Stability of rapid maxillary expansion and facemask therapy: a long-term controlled study. Am J Orthod Dentofacial Orthop. 2011 Oct;140(4):493-500. doi: 10.1016/j.ajodo.2010.09.031. PMID: 21967936. | Eligible outcomes were not measured. |
|  | Kama JD, Ozer T, Baran S. Orthodontic and orthopaedic changes associated with treatment in subjects with Class III malocclusions. Eur J Orthod. 2006 Oct;28(5):496-502. doi: 10.1093/ejo/cjl011. Epub 2006 Jun 13. PMID: 16772318. | No eligible outcome assessed |
|  | Lin HC, Chang HP, Chang HF. Treatment effects of occipitomental anchorage appliance of maxillary protraction combined with chincup traction in children with Class III malocclusion. J Formos Med Assoc. 2007 May;106(5):380-91. doi: 10.1016/S0929-6646(09)60323-5. PMID: 17561473. | No eligible outcome assessed |
|  | Westwood PV, McNamara JA Jr, Baccetti T, Franchi L, Sarver DM. Long-term effects of Class III treatment with rapid maxillary expansion and facemask therapy followed by fixed appliances. Am J Orthod Dentofacial Orthop. 2003 Mar;123(3):306-20. doi: 10.1067/mod.2003.44. PMID: 12637903. | Eligible outcomes were not measured. |
|  | Lione R, Buongiorno M, Laganà G, Cozza P, Franchi L. Early treatment of Class III malocclusion with RME and facial mask: evaluation of dentoalveolar effects on digital dental casts. Eur J Paediatr Dent. 2015 Sep;16(3):217-20. PMID: 26418925. | Eligible outcomes were not measured. |
|  | Arun T, Erverdi N. A cephalometric comparison of mandibular headgear and chin-cap appliances in orthodontic and orthopaedic view points. J Marmara Univ Dent Fac. 1994 Sep;2(1):392-8. PMID: 9582620. | Eligible outcomes were not measured. |
|  | Galeotti A, Viarani V, Franchi L, Martina S, Rongo R, D'Antò V, Uomo R, Aristei F, Festa P. Cephalometric changes of pushing splints 3 compared to rapid maxillary expansion and facemask therapy on the airway space in class III growing patients: A randomized clinical trial. Orthod Craniofac Res. 2024 Aug;27(4):552-559. doi: 10.1111/ocr.12767. Epub 2024 Feb 17. PMID: 38366756. | Eligible outcomes were not measured |
|  | Mandall N, Aleid W, Cousley R, Curran E, Caldwell S, DiBiase A, Dyer F, Littlewood S, Nute S, Campbell SJ, Atkins S, Bayoumi S, Bhatt V, Chambers P, Goodger N, Bates C, Malik O, Waring D, Bassett P. The effectiveness of bone anchored maxillary protraction (BAMP) in the management of class III skeletal malocclusion in children aged 11-14 years compared with an untreated control group: A multicentre two-arm parallel randomised controlled trial. J Orthod. 2024 Jun 6:14653125241255139. doi: 10.1177/14653125241255139. Epub ahead of print. PMID: 38845172. | Eligible outcomes were not measured |
|  | Rutili V, Souki BQ, Nieri M, Carlos ALFM, Pavoni C, Cozza P, McNamara JA Jr, Giuntini V, Franchi L. Long-term effects produced by early treatment of Class III malocclusion with rapid maxillary expansion and facemask followed by fixed appliances: A multicentre retro-prospective controlled study. Orthod Craniofac Res. 2024 Jun;27(3):429-438. doi: 10.1111/ocr.12748. Epub 2023 Dec 26. PMID: 38146808. | Eligible outcomes were not measured |
|  | Saadia M, Torres E. Sagittal changes after maxillary protraction with expansion in class III patients in the primary, mixed, and late mixed dentitions: a longitudinal retrospective study. Am J Orthod Dentofacial Orthop. 2000 Jun;117(6):669-80. PMID: 10842110. | No control group |
|  | Takada K, Petdachai S, Sakuda M. Changes in dentofacial morphology in skeletal Class III children treated by a modified maxillary protraction headgear and a chin cup: a longitudinal cephalometric appraisal. Eur J Orthod. 1993 Jun;15(3):211-21. doi: 10.1093/ejo/15.3.211. PMID: 8339762. | No control group |
|  | Bengi O, Uzel I, Işimer Y, Sağdiç D. Frankel'in "fonksiyonel regülatörü" (FR-3) ile yapilan tedavilerde sagital yöndeki sefalometrik değişkliklerin incelenmesi [In this study, it was investigated that, whether the FR-3 appliance was adequate or not in functional orthopedic therapy]. Turk Ortodonti Derg. 1989 Nov;2(2):238-47. Turkish. PMID: 2489154. | No control group |
|  | Ferro A, Cefariello S. Gli effetti della mentoniera sulla curvatura della base cranica nelle III classi scheletriche trattate con docce ed elastici intermascellari [Effects of the chin cup on curvature of the cranial base in skeletal Class III treated with splints and intermaxillary elastics]. Arch Stomatol (Napoli). 1988 Nov;29(5):977-85. Italian. PMID: 3272580. | No control group |
|  | Baik HS, Jee SH, Lee KJ, Oh TK. Treatment effects of Fränkel functional regulator III in children with class III malocclusions. Am J Orthod Dentofacial Orthop. 2004 Mar;125(3):294-301. doi: 10.1016/j.ajodo.2003.04.016. PMID: 15014405. | The sample included pseudo-class III malocclusion |
|  | Baccetti T, Tollaro I. A retrospective comparison of functional appliance treatment of Class III malocclusions in the deciduous and mixed dentitions. Eur J Orthod. 1998 Jun;20(3):309-17. doi: 10.1093/ejo/20.3.309. PMID: 9699409. | Eligible outcomes were not measured |
|  | Baccetti T, McGill JS, Franchi L, McNamara JA Jr, Tollaro I. Skeletal effects of early treatment of Class III malocclusion with maxillary expansion and face-mask therapy. Am J Orthod Dentofacial Orthop. 1998 Mar;113(3):333-43. doi: 10.1016/s0889-5406(98)70306-3. PMID: 9517727. | Eligible outcomes were not measured |
|  | Solano-Mendoza B, Iglesias-Linares A, Yañez-Vico RM, Mendoza-Mendoza A, Alió-Sanz JJ, Solano-Reina E. Maxillary protraction at early ages. The revolution of new bone anchorage appliances. J Clin Pediatr Dent. 2012 Winter;37(2):219-29. doi: 10.17796/jcpd.37.2.q0k770403v443053. PMID: 23534334. | Literature review |
|  | Klempner L. Early treatment of skeletal Class III open bite with the Tandem Appliance. J Clin Orthod. 2011 Jun;45(6):308-16; quiz 339. PMID: 21778583. | Case report |
|  | Abu Alhaija ES, Richardson A. Long-term effect of the chincap on hard and soft tissues. Eur J Orthod. 1999 Jun;21(3):291-8. doi: 10.1093/ejo/21.3.291. PMID: 10407538. | No cephalometric data at the end of functional treatment |
|  | Martin O, Muelas L, Viñas MJ. Comparative study of nasopharyngeal soft-tissue characteristics in patients with Class III malocclusion. Am J Orthod Dentofacial Orthop. 2011 Feb;139(2):242-51. doi: 10.1016/j.ajodo.2009.07.016. PMID: 21300254. | The sample included class I malocclusion in the control group. |
