# Supplementary material for: Soft tissue changes associated with Class III orthopaedic treatment in growing patients: a systematic review and meta-analysis
Source: Prog Orthod. 2025 Mar 17;26:10. doi: 10.1186/s40510-025-00558-2 (PMC11911289; doi:10.1186/s40510-025-00558-2)
Supplement: Supplementary file 3 — Supplementary Material 3 [file 40510_2025_558_MOESM3_ESM.docx]

| **Supplementary Table 3 : Risk of bias of the included RCTs** | | | | | | |
| --- | --- | --- | --- | --- | --- | --- |
| **Study** | **Randomization process** | **Deviations from intended interventions** | **Missing outcome data** | **Measurement of the outcome** | **Selection of the reported result** | **Over-all bias** |
| **Kamel 2023** | **Low risk:**  “Randomization was completed using stratifed permuted block randomization…” | **Low risk:**  Blinding of either clinician or patients was not applica- ble during the trial | **Some concerns:**  10 of 40 participants were excluded.  missing outcome data occurred for reasons that are unrelated to the outcome | **Low risk:**  The statistician was blinded during data analysis. | **Low risk:**  The protocol was not registered. However, the pre-defined outcomes mentioned in the methods section seemed reported. | **Some concerns:** |
| **Alzabibi 2021** | **Low risk:**  “Forty­ two of  these participants were randomly chosen, and then assigned randomly to the 2 study groups by us­ ing a computer­generated list of random numbers with an  allocation ratio of  1:1 …” | **Low risk:**  “Due to the nature of  the trial, blinding of  the patients and the clinicians was not applicable …” | **Low risk:**  2 participants were excluded.  missing outcome data occurred for reasons that are unrelated to the outcome | **Low risk:**  “all cepha­ lometric radiographs were coded. This ensured that the measurements were carried out by 1 assessor who was totally blinded to the study group” | **Low risk:**  “This trial was registered at Clinical.Trials.gov (NCT 03172442)”  and the outcomes mentioned in the protocol have been reported. | **Low risk.** |
| **Yavan 2021** | **Low risk:**  The 45 participants were randomly divided into three groups by a statistician using com- puter software …” | **Low risk:**  it was not possible to blind the patients and the researcher who administered the treatment; however, the researcher who performed the cephalometric measurements and the statistician who con- ducted the statistical analyses after treatment were blinded to the clinical data of the groups | **High risk:**  about 11% of the participants were lost of follow up... 6 of 51 participants.  The reason for the exclusion is somewhat unconvincing | **Low risk:**  the researcher who performed the cephalometric measurements and the statistician who con- ducted the statistical analyses after treatment were blinded to the clinical data of the groups | **Low risk:**  The protocol was not registered. However, the pre-defined outcomes mentioned in the methods section seemed reported. | **High risk** |
| **James 2020** | **Low risk:**  “Subjects were randomly allotted to group 1 or group 2 with an allocation ratio of 1:1. For the execution of planned single blinded study, blocks of random numbers were assigned using computer generated tables.” | **Low risk:**  “Blinding was possible only with regard to evaluation of cephalometric radiographs | **Low risk:**  No dropouts were reported. | **Low risk:**  “The tracings were performed randomly, by a blinded examiner,” | **Low risk:**  The protocol was not registered. However, the pre-defined outcomes mentioned in the methods section seemed reported. | **Low risk:** |
| **Celikoglu 2015** | **Low risk:**  “Thirty-four patients who met the above criteria were randomly divided into two groups using a random number table …” | **High risk:**  Due to the nature of  the trial, blinding of  the patients and the clinicians was not applicable  Two subjects excluded after intervention due to either irregular attendance or poor oral hygiene… (Post-randomization exclusions of ineligible participants). | **Some concerns:**  2of 34 participants were excluded.  missing outcome data occurred for reasons that are unrelated to the outcome | **Low risk:**  all radiographs were traced by one researcher (TU) with a random queue of the cephalometric films without knowing the group of the patient so that the researcher was blinded. | **Low risk:**  The protocol was not registered. But the pre-defined outcomes mentioned in the methods section seemed to have been reported. | **High risk** |
| **Saleh 2013** | **Low risk:**  “The randomization procedure of these 72 skeletal Class III patients was performed manu- ally by simply asking each participant to pickup a concealed opaque envelope from a black plas- tic box | **Low risk:**  Due to the nature of  the trial, blinding of  the patients and the clinicians was not applicable  “To avoid assessment bias, a blinding proce- dure for the radiographs was performed by the second and third authors” | **Low risk:**  No dropouts were reported. | **Low risk:**  “To avoid assessment bias, a blinding proce- dure for the radiographs was performed by the second and third authors” | **Low risk:**  The protocol was not registered. But the pre-defined outcomes mentioned in the methods section seemed to have been reported. | **Low risk** |
| **Vaughn 2005** | Low **risk**:  “We used a block randomization table to assign the subjects to 1 of 3 groups after obtaining proper in- formed consen” | **Low risk:**  Due to the nature of  the trial, blinding of  the patients and the clinicians was not applicable | **Some concerns:**  No details of data dropouts | **Some concerns:**  No details about assessor blinding. | **Low risk:**  The protocol was not registered. But the pre-defined outcomes mentioned in the methods section seemed to have been reported. | **Some concerns:** |
| **Kiliçoğlu and Kirliç 1998** | **Some concerns:**  No details about randomization. | **Low risk:**  Due to the nature of  the trial, blinding of  the patients and the clinicians was not applicable | **Some concerns:**  No details of data dropouts | **Some concerns:**  No details about assessor blinding. | **Low risk:**  The protocol was not registered. But the pre-defined outcomes mentioned in the methods section seemed to have been reported. | **High risk** |
