# Supplementary material for: Soft tissue changes associated with Class III orthopaedic treatment in growing patients: a systematic review and meta-analysis
Source: Prog Orthod. 2025 Mar 17;26:10. doi: 10.1186/s40510-025-00558-2 (PMC11911289; doi:10.1186/s40510-025-00558-2)
Supplement: Supplementary file 4 — Supplementary Material 4 [file 40510_2025_558_MOESM4_ESM.docx]

**Supplementary Table 4: Risk of bias assessment for the included non-randomized studies using ROBINS-I tool.**

| Study ID | Pre-intervention | | | | At-intervention | | Post-intervention | | | | | | | | Overall risk judgment |
| --- | --- | --- | --- | --- | --- | --- | --- | --- | --- | --- | --- | --- | --- | --- | --- |
|  | **Cofounding bias** | | **Selection bias** | | **Classification of intervention** | | **Bias due to deviation from intended intervention** | | **Bias due to missing data** | | **Bias in measurement of outcome** | | **Reporting bias** | |  |
|  | **judgment** | **Description** | **Judgment** | **Description** | **judgment** | **description** | **judgment** | **description** | **Judgment** | **description** | **judgment** | **description** | **judgment** | **Description** |  |
| Akbulut 2022 | **Low** | Confounder domains were measured, and were matched between the two groups  Pretreatment measurement of variables also showed no significant differences between the two groups. | **Low** | Retrospective controlled Before-after study. Sample were selected according to well-defined eligibility criteria.  All eligible participants were analyzed, and the start of intervention and follow-up were synchronized for all patients. | **low** | Intervention is well defined and intervention definition is based solely on information collected at the time of intervention. | **Low** | Deviations of the intended intervention reflects usual practice. | **Low** | Data were reasonably complete. | **Low** | Outcome measurements were comparable, accomplished by one blinded examiner. | **Low** | While unclear if authors selected the reported outcome measures a prior, the outcome measures are objective; and all  reported results correspond to all intended outcomes. | **Low** |
| Ozbilen 2022 | **Low** | Baseline characteristics were shown to be similar between the two groups; important confounding domains and pretreatment measurement of outcomes were not significantly different. | **low** | Retrospective controlled Before-after study. Sample were selected according to well-defined eligibility criteria.  All eligible participants were analyzed.  Start of intervention and start of follow up were coincided for all patients. | Low | Intervention is well-defined and based solely on information collected at time of intervention. | **Low** | There were no deviations from the intended intervention beyond what would be expected in usual practice | **low** | Data were reasonably complete. | **moderate** | Outcome measurements were comparable, accomplished by one examiner (no information if he was blinded about the exposure status), and minimally influenced by knowledge of the intervention received. | **Low** | While unclear if authors selected the reported outcome measures a prior, the outcome measures are objective; and all  reported results correspond to all intended outcomes. | **Moderate** |
| Lee 2022 | **Low** | Confounder domains were measured, and were matched between the two groups  Pretreatment measurement of variables also showed no significant differences between the two groups. | **low** | Selection of participant was according to well defined eligibility criteria, and all eligible participant were analyzed.  Start of intervention and follow up are coincided. | **low** | Intervention is well defined and intervention definition is based solely on information at time of intervention. | **low** | Deviations of the intended intervention reflects usual practice. | **low** | Data were reasonably complete.. | **moderate** | The methods of outcome assessment before and after treatment were comparable. Unclear if outcome assessors were blinded to the intervention status; this have a slight impact on the results.  Measurement error is unrelated to intervention. | **No information** | While unclear if authors selected the reported outcome measures a prior, the outcome measures are objective; and all  reported results correspond to all intended outcomes. | **moderate** |
| Lim 2021 | **Low** | Baseline characteristics were shown to be similar between the two groups; important confounding domains and pretreatment measurement of outcomes were not significantly different | **low** | subjects were selected according to well-defined eligibility criteria.  All eligible participants were included at follow-up times.  Start of intervention and start of follow up were coincided for all patients. | low | Intervention is well defined and intervention definition is based solely on information collected at the time of intervention | Low | Any deviations from intended intervention reflected usual practice. | **low** | Data were complete. | **moderate** | Outcome measurements were comparable and evaluated by one assessor; no available information if he was blinded to the treatment allocation of patients; this may have a slight impact on the results. | **low** | While unclear if authors selected the reported outcome measures a prior, the outcome measures are objective; and all reported results correspond to all intended outcomes. | **moderate** |
| Buyukcavus 2020 | **Low** | Confounder domains were measured, and were matched between the two groups  Pretreatment measurement of variables also showed no significant differences between the two groups. | **Low** | Retrospective Selection of participant was according to well defined eligibility criteria, and all eligible participant were analyzed.  Start of intervention and follow up were coincided. | **low** | Intervention is well defined and intervention definition is based solely on information at time of intervention. | **low** | Deviations of the intended intervention reflects usual practice. | **low** | Data were complete for the mcpp group at all follow up times. | **moderate** | The methods of outcome assessment were comparable. Unclear if outcome assessors were blinded to the intervention status or groups.  Measurement error is unrelated to intervention. | **low** | While unclear if authors selected the reported outcome measures a prior, the outcome measures are objective; and all  reported results correspond to all intended outcomes. | **moderate** |
| Jang 2020 | Low | Retrospective controlled before after study.  Confounder domains were measured and showed no significant differences between the two groups. | low | Patients were selected according to well defined inclusion criteria.  All eligible patients were included.  Start of intervention and start of follow up were coincided for all patients. | **low** | Intervention is well defined and intervention definition is based solely on information at time of intervention. | low | Deviations of the intended intervention reflects usual practice. | **low** | Data were complete. | **moderate** | The methods of outcome assessment were comparable. Unclear if outcome assessors were blinded to the intervention status or group ; this have a slight impact on the results.  Measurement error is unrelated to intervention. | **low** | While unclear if authors selected the reported outcome measures a prior, the outcome measures are objective; and all  reported results correspond to all intended outcomes. | **moderate** |
| Liu 2020 | **moderate** | Retrospective controlled before after study.  Confounder domains were controlled except of gender distribution; it was not reported between the groups.  Pre-treatment variables showed also no significance differences. | **low** | Patients in the two groups were selected according to well defined inclusion criteria.  All eligible patients were included.  Start of intervention and start of follow up were coincided for all patients. | **low** | Intervention is well defined and intervention definition is based solely on information at time of intervention. | **low** | Deviations of the intended intervention reflects usual practice. | **low** | Data were complete. | **moderate** | The methods of outcome assessment were comparable. Unclear if outcome assessors were blinded to the intervention status or group; this have a slight impact on the results.  Measurement error is unrelated to intervention. | **Low** | Reported results were Pre-treatment and the changes from pre- to post-treatment; post-treatment measurement were not reported.  Unclear if authors selected the reported outcome measures a prior | **moderate** |
| Sitaropoulou 2020 | **Serious** | Retrospective controlled before after study.  Confounder domains were not measured at baseline. | **low** | Retrospective Selection of participant was according to well defined eligibility criteria, and all eligible participant were analyzed.  Start of intervention and follow up were coincided. | **low** | Intervention is well defined. | **low** | Deviations of the intended intervention reflects usual practice. | **low** | Before and after treatment data were complete. | **moderate** | The methods of outcome assessment before and after treatment were comparable. Unclear if outcome assessors were blinded to the intervention status; this have a slight impact on the results.  Measurement error is unrelated to intervention. | **moderate** | Unclear if authors selected outcome measure a priori. | **serious** |
| Pavoni 2019 | **low** | Confounder domains were measured, and were matched between the two groups  Pretreatment measurement of variables also showed no significant differences between the two groups. | **Low** | Selection of participant in the two groups was according to well defined eligibility criteria, and all eligible participant were included.  Start of intervention and follow up were coincided. | **low** | Intervention is well defined and intervention definition is based solely on information at time of intervention. | **Low** | Deviations of the intended intervention reflects usual practice. | **low** | Data were reasonably complete. | **moderate** | Outcome measurements were comparable, accomplished by one examiner (no information if he was blinded about the exposure status or group); this may have a slight impact on the results | **Low** | While unclear if authors selected the reported outcome measures a prior, the outcome measures are objective; and all  reported results correspond to all intended outcomes. | **Moderate** |
| Eissa 2018 | **low** | Confounder domains were measured, and were matched between the two groups  Pretreatment measurement of variables also showed no significant differences between the two groups. | **Low** | Selection of participant in the two groups was according to well defined eligibility criteria, and all eligible participant were included.  Start of intervention and follow up were coincided. | **low** | Intervention is well defined and intervention definition is based solely on information at time of intervention. | **low** | Deviations of the intended intervention reflects usual practice. | **low** | Data were reasonably complete. | **moderate** | Outcome measurements were comparable, accomplished by one examiner. no information if he was blinded about the exposure status or group | **Low** | While unclear if authors selected the reported outcome measures a prior, the outcome measures are objective; and all  reported results correspond to all intended outcomes. | **moderate** |
| Parayaruthottam 2018 | **Serious** | Retrospective controlled before after study.  Confounder domains were not measured at baseline. | **Low** | Selection of participant in the two groups was according to well defined eligibility criteria, and all eligible participant were included.  Start of intervention and follow up were coincided. | **low** | Intervention is well defined and intervention definition is based solely on information at time of intervention. | **Low** | Deviations of the intended intervention reflects usual practice. | **low** | Data were reasonably complete. | **moderate** | Outcome measurements were comparable, accomplished by one examiner (no information if he was blinded about the exposure status or group); this may have a slight impact on the results | **moderate** | Unclear if authors selected the reported outcome measures a prior;  Study reported outcomes of cephalometric measurements. | **Serious** |
| Ağlarcı 2016 | **Low** | Confounder domains were measured, and were matched between the two groups  Pretreatment measurement of variables also showed no significant differences between the two groups. | **Low** | Selection of participant in the two groups was according to well defined eligibility criteria, and all eligible participant were included.  Start of intervention and follow up were coincided. | **low** | Intervention is well defined and intervention definition is based solely on information at time of intervention. | **low** | Deviations of the intended intervention reflects usual practice. | **low** | Data were reasonably complete. | **moderate** | Outcome measurements were comparable, accomplished by one examiner (no information if he was blinded about the exposure status or group); this may have a slight impact on the results.  Measurement error is unrelated to intervention. | **Low** | While unclear if authors selected the reported outcome measures a prior, the outcome measures are objective; and all  reported results correspond to all intended outcomes. | **Moderate** |
| Tripathi 2016 | **Serious** | Important pre-treatment measurements were not measured at baseline. | **low** | Selection of participant in the two groups was according to well defined eligibility criteria, and all eligible participant were included.  Start of intervention and follow up were coincided | **low** | Intervention is well defined and intervention definition is based solely on information at time of intervention. | **low** | Deviations of the intended intervention reflects usual practice. | **low** | Data were reasonably complete. | **moderate** | Outcome measurements were comparable, accomplished by one examiner (no information if he was blinded about the exposure status or group); this may have a slight impact on the results.  Measurement error is unrelated to intervention. | **Low** | While unclear if authors selected the reported outcome measures a prior, the outcome measures are objective; and all  reported results correspond to all intended outcomes. | **Serious** |
| Akin 2015 | **low** | Retrospective controlled before after study.  Confounder domains were measured and showed no significant differences between the two groups. | **low** | Retrospective data and images were obtained for patients according to well-defined inclusion criteria.  Images were taken before and after treatment; so start of intervention and follow up should be coincided. | **low** | Intervention is well defined and intervention definition is based solely on information at time of intervention. | **low** | Deviations of the intended intervention reflects usual practice.  (patients were treated at the same place with same operator) | **low** | No missing data;  Images were selected retrospectively before and after treatment. | **low** | Outcome measurements were comparable, accomplished by one examiner.  All personal identifiers were removed or redacted from the data .  Measurement error is unrelated to intervention. | **moderate** | outcome measures are objective; and all  reported results correspond to all intended outcomes.  unclear if authors selected the reported outcome measures a prior | **moderate** |
| Alarcón 2015 | **low** | Retrospective controlled before after study.  Confounder domains were measured and were not significantly different.  No significant differences were found between the two groups in the pre-treatment measurements of variables. | **Low** | Selection of participant in the two groups was according to well defined eligibility criteria, and all eligible participant were included.  Start of intervention and follow up were coincided. | **low** | Intervention is well defined and intervention definition is based solely on information at time of intervention. | **low** | Deviations of the intended intervention reflects usual practice. | **low** | Data were reasonably complete. | **low** | Outcome measurements were comparable, accomplished by one blinded examiner. | **Low** | While unclear if authors selected the reported outcome measures a prior, the outcome measures are objective; and all  reported results correspond to all intended outcomes. | **low** |
| Canturk and Celikoglu 2015 | **low** | Pretreatment measurement of variables showed no significant differences between  the two groups. | **low** | Selection of participant in the two groups was according to well defined eligibility criteria, and all eligible participant were included.  Start of intervention and follow up were coincided. | **low** | Intervention is well defined and intervention definition is based solely on information at time of intervention. | **low** | Deviations of the intended intervention reflects usual practice. | **low** | Data were reasonably complete. | **low** | Outcome measurements were comparable. **The researcher was blinded** | **low** | All  reported results correspond to all intended outcomes. | **low** |
| Zhao 2015 | **Serious** | Confounder domains were not measured at baseline. | **low** | Selection of participant in the two groups was according to well defined eligibility criteria, and all eligible participant were included.  Start of intervention and follow up were coincided. | **low** | Intervention is well defined and intervention definition is based solely on information at time of intervention. | **low** | Deviations of the intended intervention reflects usual practice. | **low** | Data were reasonably complete. | **Moderate** | Outcome measurements were comparable. **Unclear if the outcome assessors were blinded to the intervention status and the measurement error was not reported.** | **Moderate** | Unclear if authors selected the reported outcome measures a prior | **Serious** |
| Şar 2014 | **low** | Pretreatment measurement of variables showed no significant differences between  the two groups. | **low** | Patients were selected according to well defined eligibility criteria, all eligible patients were included at follow-up times. | **low** | Intervention is well defined and intervention definition is based solely on information at time of intervention. | **low** | Deviations of the intended intervention reflects usual practice. | **low** | Data were reasonably complete. | **Moderate** | Outcome measurements were comparable, Unclear if assessors were blinded to intervention status or group.  Measurement error was not assessment reported. | **low** | The outcome measures are objective; and all  reported results correspond to all intended outcomes. | **Moderate** |
| Lee 2012 | **low** | Retrospective controlled before after study.  Confounder domains were measured and were not significantly different.  No significant differences were found between the two groups in the pre-treatment measurements of variables.. | **moderate** | Patients were selected according to well defined eligibility criteria, all eligible patients were included at follow-up times.  Start of intervention and follow up may not be coincided for all patients. | **low** | Intervention is well defined | **low** | Deviations of the intended intervention reflects usual practice. | **low** | Data were reasonably complete. | **Moderate** | Outcome measurements were comparable, Unclear if assessors were blinded to intervention status or group.  Measurement error was not assessment reported. | **low** | The outcome measures are objective; and all  reported results correspond to all intended outcomes. | **Moderate** |
| Şar 2011 | **low** | Pretreatment measurement of variables showed no significant differences between  the two groups. | **low** | Patients were selected according to well defined eligibility criteria, all eligible patients were included at follow-up times. | **low** | Intervention is well defined and intervention definition is based solely on information at time of intervention. | **low** | Deviations of the intended intervention reflects usual practice. | **low** | Data were reasonably complete. | **Moderate** | Outcome measurements were comparable, Unclear if assessors were blinded to intervention status or group.  Measurement error was not assessment reported. | **low** | The outcome measures are objective; and all  reported results correspond to all intended outcomes. | **Moderate** |
| Cozza 2004 | **Serious** | Confounder domains were not measured at baseline. | **low** | Patients were selected according to well defined eligibility criteria, all eligible patients were included at follow-up times. | **low** | Intervention is well defined and intervention definition is based solely on information at time of intervention. | **low** | Deviations of the intended intervention reflects usual practice. | **low** | Data were reasonably complete. | **Moderate** | Outcome measurements were comparable, Unclear if assessors were blinded to intervention status or group.  Measurement error was not assessment reported. | **low** | The outcome measures are objective; and all  reported results correspond to all intended outcomes. | **Serious** |
| Üçem 2004 | **Serious** | Important pre-treatment measurements were different at baseline. | **low** | Patients were selected according to well defined eligibility criteria, all eligible patients were included at follow-up times. | **low** | Intervention is well defined and intervention definition is based solely on information at time of intervention. | **low** | Deviations of the intended intervention reflects usual practice. | **low** | Data were reasonably complete. | **Moderate** | Outcome measurements were comparable, Unclear if assessors were blinded to intervention status or group.  Measurement error was not assessment reported. | **low** | The outcome measures are objective; and all  reported results correspond to all intended outcomes. | **Serious** |
