# Supplementary material for: Soft tissue changes associated with Class III orthopaedic treatment in growing patients: a systematic review and meta-analysis
Source: Prog Orthod. 2025 Mar 17;26:10. doi: 10.1186/s40510-025-00558-2 (PMC11911289; doi:10.1186/s40510-025-00558-2)
Supplement: Supplementary file 5 — Supplementary Material 5 [file 40510_2025_558_MOESM5_ESM.docx]

**Supplementary Table 5.** A summary of quantitative measurements in each study

| Study/setting | Design | Groups | N | Pre-treatment age (years) | Outcomes | Mean ± SD | P value |
| --- | --- | --- | --- | --- | --- | --- | --- |
| ****Kamel et al. 2023**** | Rct | A: intermaxillary Class III elastics (C3E) anchored by a hybrid hyrax (HH) in the maxilla and a bone‑supported bar in the mandible  B: untreated | A: 17  B: 13 | Group 1: 11.3  Group 2: 11.5 | Ls-VRL (mm) | A: 3.15 ± 1.30  B: 1.06 ± 0.78 | < 0.001* |
|  |  |  |  |  | Li-VRL (mm) | A: − 0.24 ± 0.54  B: 1.39 ± 0.44 | < 0.001* |
|  |  |  |  |  | Pg’-VRL (mm) | A: − 0.88 ± 0.28  B: 1.53 ± 0.34 | < 0.001* |
| Akbulut et al. 2022 | NRS | A: FM + RME  B: FM + Alt-RAMEC | A: 15  B: 15 | Group 1: 11.6  Group 2: 10.9 | Nasolabial angle (deg) | A: -0.75 ± 8.44  B: –2.29 ± 9.19 | 0.635 |
|  |  |  |  |  | Ls-VRL (mm) | A: 1.57 ± 1.31  B: 2.20 ± 2.14 | 0.342 |
|  |  |  |  |  | Li-VRL (mm) | A: 0.37 ± 1.91  B: 0.15 ± 1.99 | 0.760 |
| Ozbilen et al. 2022 | NRS | A: FM + RME  B: FM + Alt-RAMEC  C: untreated | A: 15  B: 15 | Group 1: 9.94  Group 2: 9.74  Group 3: 9.46 | Upper lip | A: 1.06 ± 0.48  B: 1.22 ± 0.64  C: –0.42 ± 0.51 | A vs B: P<0.05 |
|  |  |  |  |  |  |  | B vs C: P<0.05 |
|  |  |  |  |  |  |  | A vs C: P<0.01 |
|  |  |  |  |  | Lower lip and chin | A: 0.37 ± 1.91  B: 0.15 ± 1.99 | A vs B: P<0.05 |
|  |  |  |  |  |  |  | B vs C: P<0.05 |
|  |  |  |  |  |  |  | A vs C: P<0.01 |
| Lee et al. 2022 | NRS | A: FM + MP  B: FM + RME | A: 20  B:20 | Group 1: 10.5  Group 2: 10 | Nasolabial angle (deg) | A: -1.16 ± 9.30  B: -3.11 ± 7.73 | 0.490 |
|  |  |  |  |  | Ls–Esth (mm) | A: 0.78 ± 1.46  B: 1.37 ± 1.20 | 0.214 |
|  |  |  |  |  | Li–Esth (mm) | A: -0.21 ± 1.17  B: -0.46 ± 1.12 | 0.530 |
| Alzabibi et al.  2021 | RCT | A: orthodontic removable traction appliance (ORTA)    B: untreated | A: 21  B: 19 | Group 1: 8.95  Group 2: 9.14 | Ls–Esth (mm) | A: 1.03 ±1.65  B: −0.74 ±1.79 | A vs B: P= 0.002* |
|  |  |  |  |  | Li–Esth (mm) | A: −0.80 ±1.58  B: −0.20 ±0.98 | A vs B: P= 0.166 |
|  |  |  |  |  | Nasolabial angle (deg) | A: 1.12 ±10.03  B: −0.84 ±8.20 | A vs B: P= 0.505 |
|  |  |  |  |  | Mentolabial angle (deg) | A: −3.45 ±15.39  B: 1.79 ±4.95 | A vs B: P= 0.164 |
| Lim et al. 2021 | NRS | A: creative horseshoe appliance (CHS) with two Class III elastics  B: Petit-type facemask | A: 25  B: 25 | Group 1: 8.6  Group 2: 8.9 | Nasolabial angle (deg) | A: 1.99 ± 16.2  B: - 0.99 ± 9.6 | 0.43 |
|  |  |  |  |  | Li–Esth (mm) | A: -1.14 ± 1.5  B: - 0.75 ± 1.55 | 0.366 |
| Yavan et al. 2021 | RCT | A:  B: Reverse Forsus  C: untreated | A: 15  B: 15  C: 15 | Group 1: 10.5  Group 2: 10.4  Group 3: 10.6 | Ls-VRL (mm) | A: 2.94± 2.01  B: 2.06± 1.91  C: 0.74± 0.78 | A vs B: NS |
|  |  |  |  |  |  |  | B vs C: P<0.05 |
|  |  |  |  |  |  |  | A vs C: P<0.01 |
|  |  |  |  |  | Li-VRL (mm) | A: –1.47± 2.66  B: –2.02± 3.02  C: 1.58± 1.95 | A vs B: NS |
|  |  |  |  |  |  |  | B vs C: P<0.01 |
|  |  |  |  |  |  |  | A vs C: P<0.05 |
| Buyukcavus et al. 2020 |  | A: FM/RME  B: FM/ Alt-RAMEC  C: FM/MP | A: 18  B: 19  C: 18 | Group 1: 10.5  Group 2: 11.6  Group 3: 11.9 | Ls-VRL (mm) | A: 3,07 ± 1,07  B: 3,57 ± 0,86  C: 3,83 ± 0,31 | A vs B: NS |
|  |  |  |  |  |  |  | B vs C: NS |
|  |  |  |  |  |  |  | A vs C: NS |
|  |  |  |  |  | Li-VRL (mm) | A: 0,23 ± 2,25  B: 0,15 ± 2,38  C: 0,09 ± 2,84 | A vs B: NS |
|  |  |  |  |  |  |  | B vs C: NS |
|  |  |  |  |  |  |  | A vs C: NS |
|  |  |  |  |  | Pg(s)-VRL (mm) | A: -2,06 ± 2,28  B: -1,96 ± 2,09  C: -1,84 ± 2,27 | A vs B: NS |
|  |  |  |  |  |  |  | B vs C: NS |
|  |  |  |  |  |  |  | A vs C: NS |
|  |  |  |  |  | Nasolabial angle (deg) | A: -3,72 ± 1,05  B: -3,95 ± 1,25  C: -4,83 ± 1,78 | A vs B: NS |
|  |  |  |  |  |  |  | B vs C: NS |
|  |  |  |  |  |  |  | A vs C: NS |
| James et al. 2020 | RCT | A: FM + Alt-RAMEC  B: FM + Alt-RAMEC+ Class III Elastic | A: 26  B: 26 | Group 1: 10  Group 2: 10.2 | Nasolabial angle (deg) | A: -1.21 ± 1.26  B: ‑4.06 ± 3.64 | 0.001 |
|  |  |  |  |  | Ls–Esth (mm) | A: 1.76± 1.83  B: 4.33 ± 2.65 | 0.002 |
|  |  |  |  |  | Li–Esth (mm) | A: -0.60 ± 1.65  B: ‑1.38 ± 1.49 | 0.079 |
|  |  |  |  |  | Pg(s)-VRL (mm) | A: -2.88 ± 3.01  B: ‑2.45 ± 1.70 | 0.522 |
| Jang et al. 2020 | NRS | A: Skeletal- anchored facemasks (SAFM)  B: tooth-borne facemasks (TBFM) | A: 31  B: 36 | Group 1: 11.1  Group 2: 11 | Nasolabial angle (deg) | A: 3.10 ± 8.12  B: -1.10 ± 8.02 | 0.038* |
|  |  |  |  |  | Ls-VRL (mm) | A: 4.72 ± 2.76  B: 3.13 ± 2.13 | 0.021* |
|  |  |  |  |  | Nasofacial angle (deg) | A: 0.50 ± 5.53  B: 2.06 ± 3.43 | 0.153 |
| Liu et al. 2020 |  | A: FM/ Banded appliance  B: FM/ Modiﬁed appliance | A: 20  B: 20 | Group 1: 8.35  Group 2: 8.65 | Ls-VRL (mm) | A: 2.36 ± 0.59  B: 2.45 ± 0.91 | NS |
|  |  |  |  |  | Li-VRL (mm) | A: –0.51 ± 0.59  B: - 1.65 ± 1.10 | NS |
|  |  |  |  |  | Pg(s)-VRL (mm) | A: -2.76 ± 0.63  B: -4.78 ± 0.95 | P<0.05 |
| Sitaropoulou et al. 2020 | NRS | A: FM + Alt-RAMEC  B: untreated | A: 20  B: 16 | Group 1: 9.74  Group 2: 9.44 | Labiale superior | A: 2.66± 1.82  B: 0.2± 1.11 | p< 0.01 |
|  |  |  |  |  | Subalare r-l | A: 1.71± 2.34 B: 0.14± 1.44 | p< 0.05 |
|  |  |  |  |  | Alar curvature r-l | A: 1.96± 1.4  B: 0.31± 0.6 | p< 0.01 |
| Pavoni et al. 2019 | NRS | A: FM/RPE  B: untreated | A: 32  B: 20 | Group 1: 8.4  Group 2: 8.7 | UL-SL (mm) | FM: 1.7±1.7  C: 0.0 ±1.4 | 0.001 |
|  |  |  |  |  | LL-SL (mm) | FM: 0.0 ±1.4  C: 0.8 ±1.4 | 0.036 |
|  |  |  |  |  | Nasolabial angle (deg) | FM: -4.3 ±6.7  C: 0.3 ±8.5 | 0.033 |
|  |  |  |  |  | Profile facial angle (deg) | FM: -3.6 ±4.3  C: 0.8 ±2.1 | 0.000 |
|  |  |  |  |  | Mandibular sulcus (deg) | FM: - 4.4 ±4.0  C: 1.4 ±3.4 | 0.000 |
| Eissa et al. 2018 | NRS | A: miniscrew-anchored inverted (FRD)  B: untreated | A: 16  B: 16 | Group 1: 12.3  Group 2: 11.9 | Ls–Esth (mm) | A: 0.95 ± 0.74  B: - 0.07 ± 0.38 | .001 |
|  |  |  |  |  | Li–Esth (mm) | A: -1.56 ± 0.73  B: 0.15 ± 0.46 | .000 |
|  |  |  |  |  | Nasolabial angle (deg) | A: -3.68 ± 1.33  B: 0.29 ± 1.58 | .000 |
| Parayaruthottam et al. 2018 | NRS | A: FM + RME  B: FM + Alt-RAMEC | A: 9  B: 9 | Group 1: 10.1  Group 2: 10.3 | Nasolabial angle (deg) | A: 1.26 ± 5.15  B: −0.31 ± 7.89 | Ns |
|  |  |  |  |  | Ls-VRL (mm) | A: 1.62 ± 2.01  B: 3.39 ± 1.45 | P<0.01 |
|  |  |  |  |  | Li-VRL (mm) | A: -1.37 ± 2.73  B: - 0.71 ± 1.38 | Ns |
|  |  |  |  |  | Pg(s)-VRL (mm) | A: - 2.89 ± 2.28  B: - 2.87 ± 2.27 | Ns |
| Ag˘larcı et al. 2016 | NRS | A: FM  B: SA Skeletal- anchored | A: 25  B: 25 | Group 1: 11.2  Group 2: 11.7 | Nasolabial angle (deg) | A: -0.41 ± 13.4  B: 3.48 ± 9.64 | 0.245 |
|  |  |  |  |  | Ls-VRL (mm) | A: 2.4 ± 2.99  B: 3.57 ± 2.19 | 0.136 |
|  |  |  |  |  | Li-VRL (mm) | A: - 0.89 ± 2.71  B: 0.93 ± 3.66 | 0.061 |
| Tripathi et al. 2016 | NRS | A: skeletal anchored maxillary protraction (SAMP)  B: Conventional facemask/ RME | A: 10  B:10 | Group 1: 10.1  Group 2: 9.9 | UL-VP (mm) | A: 2.60±1.07  B: 2.40±1.71 | 0.684 |
|  |  |  |  |  | LL-VP (mm) | A: −0.20±1.68  B: −0.20±2.85 | 0.684 |
|  |  |  |  |  | Pg’-VP (mm) | A: −1.80±1.68  B: −3.20±3.08 | 0.052 |
| Akin et al. 2015 | NRS | A: FM/RPE  B: CC  C: untreated | A: 25  B: 25  C: 17 | Group 1: 10.3  Group 2: 9.8  Group 3: 10.1 | Ls–Esth (mm) | A: +3.76±1.48  B: +2.91±2.01  C: +0.65±1.03 | A vs B: NS |
|  |  |  |  |  |  |  | B vs C: P<0.05 |
|  |  |  |  |  |  |  | A vs C: P<0.001 |
|  |  |  |  |  | Li–Esth (mm) | A: −0.15±0.93  B: −1.50±2.12  C: +0.28±1.58 | A vs B: NS |
|  |  |  |  |  |  |  | B vs C: NS |
|  |  |  |  |  |  |  | A vs C: NS |
| Alarcón et al. 2015 | NRS | A: CC  B: untreated | A: 42  B: 25 | Group 1: 8.5  Group 2: 8.5 | Nasolabial angle (deg) | A: −2.57± 17  B: 2.92 ± 15.8 | P= 0.177 |
|  |  |  |  |  | Facial convexity angle | A: -5.09 ± 4.2  B: -0.64 ± 4.8 | <0.001 |
|  |  |  |  |  | Ls–Esth (mm) | A: -0.81 ± 1.95  B: 1.20 ± 1.64 | <0.001 |
|  |  |  |  |  | Li–Esth (mm) | A: 0.24 ± 2.18  B: −0.04 ± 1.79 | 0.702 |
|  |  |  |  |  | Pg’-GD (mm) | A: -1.21 ± 3.14  B: 4.76 ± 4.11 | <0.001 |
|  |  |  |  |  | Upper lip thickness (mm) | A: 0.31± 1.63  B: 0.88 ± 1.62 | 0.236 |
|  |  |  |  |  | Lower lip thickness (mm) | A: 0.38 ± 1.54  B: 0.76 ± 2.37 | 0.628 |
| Canturk and Celikoglu 2015 | NRS | A: FM started after the completion of the Alt-RAMEC  B: FM started simultaneously with the Alt-RAMEC | A: 15  B:15 | Group 1: 11.2  Group 2: 10.5 | Ls-VRL (mm) | A: 3.40 ± 1.90  B: 2.83± 2.54 | .517 |
|  |  |  |  |  | Li-VRL (mm) | A: -0.93 ± 2.82  B: -0.46 ± 2.79 | .667 |
|  |  |  |  |  | Pg(s)-VRL (mm) | A: -1.81± 1.77  B: - 2.28 ± 2.33 | .564 |
|  |  |  |  |  | Convexity Angle (deg) | A: 8.32 ± 2.88  B: 8.52 ± 2.57 | .850 |
| Celikoglu et al. 2015 | RCT | A: FM + RME  B: Mini maxillary protractor (MMP) | A: 16  B:16 | Group 1: 12  Group 2: 11.5 | Ls–Esth (mm) | A: -1.38 ± 1.20  B: −2.60± 1.55 | 0.031 |
|  |  |  |  |  | Li–Esth (mm) | A: -0.96 ± 1.40  B: −0.40 ± 1.31 | 0.278 |
|  |  |  |  |  | Nasolabial angle (deg) | A: 1.03 ± 8.50  B: −3.38 ± 10.13 | 0.227 |
|  |  |  |  |  | Facial angle (deg) | A: -4.49 ± 1.75  B: −4.65 ± 4.23 | 0.877 |
| Sar et al. 2014 | NRS | A: FM+MP  B: EL+MP  C: untreated | A: 17  B: 17  C: 17 | Group 1: 11.2  Group 2: 11.2  Group 3: 9.9 | Ls-VRL (mm) | A: 3.55 ± 1.49  B: 3.61 ± 1.86  C: 1.08±1 | A vs B: NS |
|  |  |  |  |  |  |  | B vs C: P<0.05 |
|  |  |  |  |  |  |  | A vs C: P<0.05 |
|  |  |  |  |  | Li-VRL (mm) | A: -1.5±1.88  B: -1.55±2.17  C: 1.79±1.07 | A vs B: NS |
|  |  |  |  |  |  |  | B vs C: NS |
|  |  |  |  |  |  |  | A vs C: NS |
|  |  |  |  |  | Pg’-VRL (mm) | A: -3.05 ±2.03  B: -3.29 ±1.93  C: 2.02 ±0.99 | A vs B: NS |
|  |  |  |  |  |  |  | B vs C: P<0.001 |
|  |  |  |  |  |  |  | A vs C: P<0.001 |
|  |  |  |  |  | Sn-Me’ (mm) | A: 2.29 ±2.22  B: 4.08 ±1.80  C: 0.14 ±1.56 | A vs B: P<0.01 |
|  |  |  |  |  |  |  | B vs C: NS |
|  |  |  |  |  |  |  | A vs C: NS |
| Saleh et al. 2013 | RCT | A: Removable mandibular retractor (RMR) appliance.  B: untreated | A: 33  B: 34 | Group 1: 7.5  Group 2: 7.3 | Is-TV (mm) | A: 4.99 ± 0.99  B: 0.99 ± 0.48 | A vs B: P<0.001* |
|  |  |  |  |  | Ii-TV (mm) | A: 0.35 ± 0.58  B: 1.56 ± 0.57 | A vs B: P<0.001* |
|  |  |  |  |  | Nasolabial angle (deg) | A: -8.87 ± 1.92  B: 2.16 ± 1.39 | A vs B: P<0.001* |
|  |  |  |  |  | Mentolabial angle (deg) | A: 5.24 ± 1.20  B: 0.58 ± 1.54 | A vs B: P<0.001* |
|  |  |  |  |  | Profile facial angle (deg) | A: 5.13 ± 1.52  B: 0.11 ± 0.67 | A vs B: P<0.001* |
|  |  |  |  |  | Pg’-TV (mm) | A: 1.48 ±0.78  B: 3.38 ±0.94 | A vs B: P<0.001* |
| Lee et al. 2012 | NRS | A: FM + MP  B: FM + RME | A: 10  B:10 | Group 1: 11.2  Group 2: 10.7 | Ls–Esth (mm) | A: 1.79 ± 2.26  B: 2.55 ± 1.36 | 0.3743 |
|  |  |  |  |  | Li–Esth (mm) | A: 0.18 ± 2.30  B: -0.41± 0.89 | .4596 |
| Sar et al. 2011 | NRS | A: FM +MP  B: FM/RPE  C: untreated | A: 15  B: 15  C: 15 | Group 1: 10.9  Group 2: 10.3  Group 3: 10 | Ls-VRL (mm) | A: 3.43 ±1.76  B: 2.63 ±2.7  C: 0.83 ± 1.09 | A vs B: P<0.001 |
|  |  |  |  |  |  |  | B vs C: P<0.001 |
|  |  |  |  |  |  |  | A vs C: P<0.001 |
|  |  |  |  |  | Li-VRL (mm) | A: -1.33 ± 2.12  B: -1.16 ±2.41  C: 1.6 ±1.05 | A vs B: NS |
|  |  |  |  |  |  |  | B vs C: P<0.001 |
|  |  |  |  |  |  |  | A vs C: P<0.001 |
|  |  |  |  |  | Pg’-VR (mm) | A: -2.66 ± 2.34  B: -2.53 ± 2.23  C: 1.76 ±1.37 | A vs B: NS |
|  |  |  |  |  |  |  | B vs C: P<0.001 |
|  |  |  |  |  |  |  | A vs C: P<0.001 |
|  |  |  |  |  | Sn-Me’ (mm) | A: 2.43 ±2.08  B: 3.86 ±2.23  C: 0.30 ±1.46 | A vs B: P<0.001 |
|  |  |  |  |  |  |  | B vs C: P<0.001 |
|  |  |  |  |  |  |  | A vs C: P<0.001 |
| Vaughn et al. 2005 | RCT | A: RME+FM  B: FM  C: untreated | A: 15  B: 14  C: 17 | Group 1: 7.4  Group 2: 8.1  Group 3: 6.6 | Nasolabial angle (deg) | A: 1.64 ±5.9  B: 5.63 ± 6.2  C: -3.2 ± 5.9 | A vs B: NS |
|  |  |  |  |  |  |  | B vs C: P<0.01 |
|  |  |  |  |  |  |  | A vs C: P < 0.05 |
| Cozza et al. 2004 | NRS | A: Facemask + Bionator III (FM+BIO)  B: untreated | A: 30  B: 24 | Group 1: 6.8  Group 2: 6.9 | Ls–Esth (mm) | A: 2.92 ±1.15  B: -0.23 ±0.6 | A vs B: P<0.01* |
|  |  |  |  |  | Li–Esth (mm) | A: 0.15 ± 1.98  B: 0.69 ± 0.6 | A vs B NS |
|  |  |  |  |  | Nasolabial angle (deg) | A: -3.11± 11.13  B: 1.04± 0.83 | A vs B NS |
|  |  |  |  |  | Convexity Angle (deg) | A: -5.23 ± 3.85  B: 1.46 ± 0.85 | A vs B: P<0.01* |
|  |  |  |  |  | Upper lip thickness (mm) | A: 1.65±2.21  B: -0.50 ±0.50 | A vs B: P<0.01* |
|  |  |  |  |  | Upper lip strain (mm) | A: 0.85±2.14  B: -0.88 ±0.74 | A vs B: P<0.01* |
| Ucem et al. 2004 | NRS | A: Double-plate appliance (DPA) with 2 Class III elastics  B: FM  C: untreated | A: 14  B: 14  C: 14 | Group 1: 10.3  Group 2: 10.5  Group 3: 9.8 | Nasolabial angle (deg) | A: -2.5 ± 2.68  B: 0 ± 3.19  C: 3.3 ± 1.86 | A vs B: NS |
|  |  |  |  |  |  |  | B vs C: NS |
|  |  |  |  |  |  |  | A vs C: NS |
|  |  |  |  |  | Li–Esth (mm) | A: -1.1 ± 0.34  B: -0.2 ± 0.47  C: 0.1 ± 0.59 | A vs B: NS |
|  |  |  |  |  |  |  | B vs C: NS |
|  |  |  |  |  |  |  | A vs C: NS |
| Kiliçoğlu and Kirliç 1998 | RCT | A: FM  B: untreated | A: 16  B: 16 | Group 1: 8.6  Group 2: 9.2 | Ls-VRL (mm) | A: 4.5 ± 2.5  B: 1.9 ± 2.7 | < 0.05 |
|  |  |  |  |  | Li-VRL (mm) | A: 1.1 ±2.89  B: 2.1 ±3.05 | NR |
|  |  |  |  |  | Profile facial angle (deg) | A: -1.81 ± 2.43  B: 1.6 ± 2.05 | < 0.001 |
|  |  |  |  |  | Nasolabial angle (deg) | A: -4.16 ± 10.64  B: -7.15 ± 11.92 | NR |
|  |  |  |  |  | Mentolabial angle (deg) | A: -4.72 ±20.11  B: -0.55 ±16.28 | NR |
|  |  |  |  |  | Upper lip thickness (mm) | A: -0.84 ± 1.98  B: 0.30 ± 1,69 | NR |
|  |  |  |  |  | Upper lip strain (mm) | A: -0.25 ± 2.77  B: 0.45 ± 1.0 | NR |
|  |  |  |  |  | PgS-VRL (mm) | A: -0.34 ± 2.00  B: 2.70 ± 2.00 | < 0.001 |
